# MicroRNA-1 Expression and Function in *Hyalomma Anatolicum anatolicum* (Acari: Ixodidae) Ticks

**DOI:** 10.3389/fphys.2021.596289

**Published:** 2021-04-08

**Authors:** Jin Luo, Qiaoyun Ren, Wenge Liu, Xiaofei Qiu, Gaofeng Zhang, Yangchun Tan, Runlai Cao, Hong Yin, Jianxun Luo, Xiangrui Li, Guangyuan Liu

**Affiliations:** ^1^State Key Laboratory of Veterinary Etiological Biology, Key Laboratory of Veterinary Parasitology of Gansu Province, Lanzhou Veterinary Research Institute, Chinese Academy of Agricultural Sciences, Lanzhou, China; ^2^Ministry of Education (MOE) Joint International Research Laboratory of Animal Health and Food Safety, College of Veterinary Medicine, Nanjing Agricultural University, Nanjing, China; ^3^Jiangsu Co-Innovation Center for the Prevention and Control of Important Animal Infectious Disease and Zoonose, Yangzhou University, Yangzhou, China

**Keywords:** ticks, *Hyalomma anatolicum anatolicum*, microRNA, miR-1, biological function

## Abstract

MicroRNAs act as mRNA post-transcriptional regulators, playing important roles in cell differentiation, transcriptional regulation, growth, and development. In this study, microRNA expression profiles of *Hyalomma anatolicum anatolicum* ticks at different developmental stages were detected by high-throughput sequencing and functionally assessed. In total, 2,585,169, 1,252,678, 1,558,217, and 1,155,283 unique reads were obtained from eggs, larvae, nymphs, and adults, respectively, with 42, 46, 45, and 41 conserved microRNAs in these stages, respectively. Using eggs as a control, 48, 43, and 39 microRNAs were upregulated, and 3, 10, and 9 were downregulated in larvae, nymphs, and adults, respectively. MicroRNA-1 (miR-1) was expressed in high abundance throughout *Ha. anatolicum* development, with an average of nearly one million transcripts, and it is highly conserved among tick species. Quantitative real-time PCR (qPCR) showed that miR-1 expression gradually increased with tick development, reaching the highest level at engorgement. Differential tissue expression was detected, with significantly higher levels in the salivary glands and epidermis than in the midgut. Inhibition assays showed no significant change in body weight or spawning time or amount between experimental and control groups, but there was a significant difference (*p* < 0.01) in engorgement time. With miR-1 inhibition, ticks displayed obvious deformities during later development. To more fully explain the microRNA mechanism of action, the miR-1 cluster was analyzed according to the target gene; members that jointly act on Hsp60 include miR-5, miR-994, miR-969, and miR-1011. Therefore, microRNAs are critical for normal tick development, and the primary structure of the mature sequence of miR-1 is highly conserved. Nonetheless, different developmental stages and tissues show different expression patterns, with a certain role in prolonging feeding. miR-1, together with other cluster members, regulates mRNA function and may be used as a molecular marker for species origin, evolution analysis, and internal reference gene selection.

## Introduction

*Hyalomma anatolicum anatolicum* belongs to the taxa Parasitiformes, Ixodoidea, Ixodidae, and *Hyalomma*. At present, ticks are only reported in the semidesert areas of Xinjiang Uyghur Autonomous Region in China (Ghosh et al., 2017). However, ticks are distributed worldwide, including the former Soviet Union, India, Nepal, Pakistan and Central Asia, North Africa, and in eastern European countries. Ticks parasitize cattle, sheep, camels, horses, donkeys, and a few wild animals; serve as a transmission vector of the *Crimean–Congo hemorrhagic fever* (Xinjiang hemorrhagic fever); can be naturally infected with *Coxiella burnetii* (Q fever); and can spread brucellosis and piriformis disease (Luo et al., 2003; Guan et al., 2009). Ticks cause serious harm to the livestock industry and public health. Therefore, the quest for vaccine candidates to control these arthropod vectors remains a pivotal and rational approach to controlling these diseases. The 4D8 tick protective antigen has shown promising results in controlling ixodid tick infestations (Ghosh et al., 2019).

As key components of most regulatory events, microRNAs play important roles at the post-transcriptional level in various developmental and physiological processes (Bartel, 2004; Lewis et al., 2005; Zhang et al., 2013; Meng et al., 2014; Luo et al., 2015, 2019; Wang et al., 2016; Xie et al., 2016). MicroRNA-1 (miR-1) is another important microRNA molecule discovered in recent years. Its precursor molecules regulate target genes in cells. Studies have confirmed two subtypes of mature miR-1 (miR-1-1 and miR-1-2) in primates. These microRNAs play an important role in skeletal and cardiac muscle development (Lauschke et al., 2016; Metlapally et al., 2016). In particular, miR-1 is crucial in the development of cardiac hypertrophy, myocardial infarction, arrhythmia, and other cardiac diseases (Jeong et al., 2016; Klingelhöffer et al., 2016; Zhou et al., 2017). Downregulation of miR-1 has been widely used as a biomolecular marker for myocardial infarction (Mishima et al., 2007; Zhao et al., 2007; Silvestri et al., 2009; Zorio et al., 2009; Bostjancic et al., 2010; Cai et al., 2010). Other studies have shown that the heat shock protein 60 (Hsp60) is a target gene of miR-1.

Hsp60 is a component of the defense mechanism against diabetic myocardial injury. The expression level of Hsp60 is significantly decreased in diabetic myocardial injury tissues. *In vivo* and *in vitro* experiments confirm that increased glucose levels in atrophic cardiomyocyte cells lead to upregulation of miR-1 expression, which accelerates glucose-mediated apoptosis by Hsp60 in cardiomyocytes (D’Alessandra et al., 2010). miR-1 also plays an important role in the differentiation and development of smooth muscle and skeletal muscle cells (Chen et al., 2010, 2011; Shan et al., 2010). For example, miR-1 was found to be a factor in specifically differentiated smooth muscle cells isolated from embryonic stem cell-derived cultures. Loss of miR-1 function can cause a decrease in smooth muscle biomarkers and the number of derived smooth muscle cells. Indeed, evidence has shown that miR-1 is the main factor regulating smooth muscle cell differentiation, and Kruppel-like 4 (KLF4) is affected by miR-1 downregulation. The recognition site of miR-1 is located in the 3′-UTR of KLF4, and inhibition of miR-1 reduces KLF4 expression and smooth muscle cell differentiation (Townley-Tilson et al., 2010).

Mutation of mir-1 and mir-206 sites in the 3′-UTR of muscle growth inhibition factor have been established in the Texel sheep model, leading to changes in muscle phenotype (Xie et al., 2010). Therefore, it is important to understand miR-1 function by examining the targets of its microRNA cluster. There are 142 target genes of the miR-1 cluster based on Pictar software and 187 conserved sites and 14 non-conserved sites with 181 conserved genes according to TargetScanFly. Gene Ontology enrichment analysis shows that target genes of the miR-1 cluster are involved in various biological processes, including gene expression regulation, nucleic acid metabolism, cell communication, cell division, growth, and proliferation. Therefore, studying the function of target genes regulated by miR-1 in genetic interaction networks is of great importance for better understanding the mechanisms of certain diseases and for drug development.

miR-1 plays an important regulatory role in mammalian muscles, but its function in ticks remains unclear. Here, the expression level of miR-1 in different developmental stages and tissues of ticks is analyzed to predict its possible biological functions. An inhibitor of miR-1 was injected into the fourth sarcomere, and physiological indicators were assessed at various developmental stages. The molecular mechanisms were also investigated, and the biological function of miR-1 in tick development was explored. This is the first report on the function of miR-1 in ticks, and we present the characteristics of tick development upon abnormal expression of miR-1. This study provides new insight into the function of microRNAs in ticks as well as a theoretical basis for the prevention and control of ticks.

## Materials and Methods

### Ethics Statement

The present study was approved by the Ethics Committee of the Lanzhou Veterinary Research Institute, Chinese Academy of Agricultural Sciences (approval no. LVRIAEC 2019-006), and the *Ha. anatolicum* samples were collected in strict accordance with the requirements of the Ethics Procedures and Guidelines of the People’s Republic of China.

### Tick Collection and RNA Extraction

In this study, *Ha. anatolicum* ticks were obtained from Xinjiang Uyghur Autonomous Region (XUAR) and identified using morphology by the Animal Research Institute (Lanzhou Veterinary Research Institute). The ticks were cultured with feeding on rabbits during various developmental stages in the laboratory. After cultivation, eggs were collected into sterile tubes for incubation. Approximately 2 g of larvae, on average, was divided into two parts. One batch was obtained for microRNA extraction. The other batch was placed in a mesh bag attached to a host for 27 days until the unfed nymphs and unfed adult ticks were collected. To remove external bacteria, the collected ticks were immediately placed in phosphate-buffered saline (PBS) and washed twice in a solution containing 0.133 M NaCl, 1.11% sodium dodecyl sulfate (SDS), and 0.0088 M ethylenediaminetetraacetic acid (EDTA).

To analyze the dynamic characteristics of microRNAs from different developmental stages of *Ha*. *anatolicum*, four samples (eggs, larvae, nymphs, and adults) were homogenized by freezing in liquid nitrogen and grinding into a powder using a sterile mortar and pestle. Total RNA and/or enriched small RNA (sRNA) fractions were isolated from whole-tick samples using the miScript microRNA isolation kit according to the manufacturer’s instructions (QIAGEN, China). The quantity and integrity of the total RNA were assessed using an Agilent 2100 Bioanalyzer system (Agilent Technologies, United States). Total RNA was stored at −80°C until use.

### Small RNA Isolation and High-Throughput Sequencing

The quality of total RNA was analyzed using a Shimadzu 206-97213C BioSpec-nano analyzer system with denaturing polyacrylamide gel electrophoresis. An sRNA library was generated according to the Illumina sample preparation instructions (Clop et al., 2006). Briefly, total RNA samples were size fractionated on a 15% Tris-borate-EDTA-urea polyacrylamide gel. RNA fragments 18–50 nt long were isolated, quantified, and ethanol precipitated. A 5′ adapter (Illumina) was ligated to the RNA fragments with T4 RNA ligase (Promega). The ligated RNAs were size fractionated on a 15% Tris-borate-EDTA-urea polyacrylamide gel, and 41–76-nt-long RNA fragments were isolated. Next, 3′-adapter (Illumina) ligation was performed, followed by a second size fractionation using the same gel conditions as described above. The 64–99-nt-long RNA fragments were isolated by gel elution and ethanol precipitation. The ligated RNA fragments were reverse transcribed to single-stranded cDNAs using M-MuLV (Invitrogen) with RT primers (as recommended by Illumina). cDNAs were amplified with pfx DNA polymerase (Invitrogen) using 20 PCR cycles and the Illumina sRNA primer set. The PCR products were purified on a 12% Tris-borate-EDTA polyacrylamide gel, and a slice containing cDNAs of 80∼115 bp was excised. This fraction was eluted, and the recovered cDNAs were precipitated and quantified using a NanoDrop instrument (Thermo Fisher Scientific) and TBS-380 minifluorometer (Turner Biosystems) with PicoGreenH dsDNA quantization reagent (Invitrogen). The concentration of the sample was adjusted to 10 nM, and 10 μl was used for sequencing. The purified cDNA library was used for cluster generation (with the Illumina Cluster Station) and then sequenced using a HiSeq2000 following the manufacturer’s instructions.

### Small RNA Bioinformatics Analysis

Sequence data (raw data or raw reads) conversion was conducted by base calling. We used software developed by BGI for HiSeq sequencing data processing, eliminating some contaminants and low-quality reads to obtain final clean reads. The data were processed according to the following steps: (1) removing low-quality reads; (2) removing reads with 5′ primer contamination; (3) removing reads without a 3′ primer; (4) removing reads without the insert tag; (5) removing reads with poly-A; (6) removing reads shorter than 18 nt; (7) summarizing the length distribution of the clean reads. Normally, the length of an sRNA is between 18 and 30 nt, and length distribution analysis is helpful to assess the length compositions of an sRNA sample. For example, microRNA is normally 21 or 22 nt, siRNA is 24 nt, and piRNA is 30 nt. The clean read data were assembled using SOAPdenovo short sequence assembly software^1^ and used to assemble sRNAs for mapping to the *Ixodes scapularis* genome by the BowTie software^2^ and SOAP assembly (Hafner et al., 2008). sRNAs were aligned to the microRNA precursor of corresponding species (the mature microRNA if no precursor information for that species was found in miRBase21) to obtain the microRNA count as well as base bias at the first position of the identified microRNAs with certain lengths and for each position of all identified microRNAs. The sRNA tags were annotated as rRNA, scRNA, snoRNA, snRNA, or tRNA using GenBank and Rfam databases with Tag2 annotation software (developed by BGI). In the above alignment and annotation, some sRNA tags may be mapped to more than one category. Thus, to ensure unique sRNAs mapped to only one annotation, we followed the following priority rule: rRNAetc (in which GenBank > Rfam) > known microRNA > repeat > exon > intron (Li et al., 2008). The total rRNA proportion is a marker of sample quality control, whereby high-quality samples should be less than 60% for plants and 40% for animals. The unannotated sequences were used to predict potential novel microRNA candidates.

The characteristic hairpin structure of the microRNA precursor can be used to predict novel microRNAs (Calabrese et al., 2007). BGI developed the prediction software Mireap to predict novel microRNA by exploring the secondary structure, Dicer cleavage site, and minimum free energy of the unannotated sRNA tags mapped to a genome. Mireap can be accessed at http://sourceforge.net/projects/mireap/ (Zuker, 2003) under the following parameter settings according to Zuker and Jacobson (Li et al., 2012): minimal microRNA sequence length 18; maximal microRNA sequence length 26; minimal microRNA reference sequence length 20; maximal microRNA reference sequence length 24; minimal depth of Drosha/Dicer cutting site 3; maximal copy number of microRNAs on reference 20; maximal free energy allowed for a microRNA precursor −18 kcal/mol; maximal space between microRNA and microRNA^∗^ 35; minimal base pairs of microRNA and microRNA^∗^ 14; maximal bulge of microRNA and microRNA^∗^ 4; maximal asymmetry of microRNA/microRNA^∗^ duplex 5; flanking sequence length of microRNA precursor 10. Stem-loop hairpins were considered typical in accordance with the following three criteria (Li et al., 2012): mature microRNAs were present in one arm of the hairpin precursors, which lacked large internal loops or bulges; the secondary structures of the hairpins were stable, with a free energy of hybridization lower than −18 kcal/mol; and hairpins were located in intergenic regions or introns. Genes with sequences and structures that fulfilled the three criteria, forming perfect stem-loop structures, were considered microRNA candidates. Finally, all remaining novel microRNA candidates were subjected to MiPred^3^ to filter out pseudopremicroRNAs using the following settings: minimum free energy >−20 kcal/mol or *P* > 0.05 (Zuker and Jacobson, 1998).

### Prediction of microRNA Targets and Gene Ontology (GO) Analysis

Because no 3′-UTR database is currently available, putative target genes of novel microRNA candidates were explored by aligning microRNA sequences with the tick EST database in NCBI. The rules used for target prediction were based on those suggested by Allen et al. (2005) and Schwab et al. (2005), as follows: (1) no more than four mismatches between sRNA and target (G-U bases count as 0.5 mismatches); (2) no more than two adjacent mismatches in the microRNA/target duplex; (3) no adjacent mismatches in positions 2–12 of the microRNA/target duplex (5′ of microRNA); (4) no mismatches in positions 10–11 of the microRNA/target duplex; (5) no more than 2.5 mismatches in positions 1–12 of the microRNA/target duplex (5′ of microRNA); and (6) minimum free energy (MFE) of the microRNA/target duplex ≥75% of the MFE of the microRNA bound to its perfect complement. More strictly, no more than two mismatches between the microRNA sequence and potential microRNA target were allowed.

Gene Ontology (GO) is an international standardized classification system for gene function that supplies a set of controlled vocabulary to comprehensively describe the properties of genes and gene products. There are three ontologies in GO: molecular function, cellular component, and biological process. GO terms significantly enriched for the predicted target gene candidates of microRNAs compared with the reference gene background and the genes corresponding to certain biological functions were analyzed. This method first maps all target gene candidates to GO terms in the database^4^ (Peng et al., 2009), calculating gene numbers for each term, and then applies a hypergeometric test to find significantly enriched GO terms for target gene candidates compared with the reference gene background.

### Real-Time Quantitative Polymerase Chain Reaction

Stem-loop real-time reverse transcription polymerase chain reaction (RT-PCR) with SYBR Green was used for the analysis of microRNA expression in *Ha*. *anatolicum* according to the manufacturer’s protocol. A stem-loop forward primer (5′-GTC GTA TCC AGT GCA GGG TCC GAG GTA TTC GCA CTG GAT ACG AC-3′) was used to quantify microRNA expression because it can provide more specificity and sensitivity than linear primers. The reverse universal primer (10× miScript Universal primer) was provided by QIAGEN Co, Ltd, China, and the forward primer was designed by Primer Premier 5.0 (miR1F: 5′-TCC GTT CGG ATC ACC GTG CTT C-3′). The β-*Actin* (EF488512) gene was designed as a reference gene with the following primers used: sense primer, 5′-TGT GAC GAC GAG GTT GCC G-3′; anti-sense primer, 5′-GAA GCA CTT GAG GTG GAC AAT G-3′. All forward primers were synthesized by Shenggong Co. Ltd., China (Table 1). Real-time quantitative PCR was performed using an M × 3000pTM SYBR Green real-time quantitative PCR analyzer (QIAGEN Biotechnology Co., Ltd., China). Briefly, 2 μg of microRNA was reverse transcribed using a miScript II microRNA cDNA Synthesis Kit (QIAGEN Biotechnology Co., Ltd., China). The reverse transcription reaction system included 4 μl of 5 × miScript HiFlex Buffer, 2 μl of 10 × miScript Nucleics Mix, 2 μl of miScript Reverse Transcriptase Mix and RNase-free dH_2_O to a final volume of 20 μl. The RT-PCR program was set to 37°C for 60 min followed by 95°C for 5 min. The cDNA products were stored at −20°C. Relative real-time quantitative PCR was performed with a miScript SYBR Green PCR kit (QIAGEN Biotechnology Co., Ltd., China). The reaction solution was prepared on ice and comprised 10 μl of 2 × QuantiTect SYBR Green PCR Master Mix, 2 μl of 2 × miScript Universal primer (10 μM), 2 μl of forward primer (10 μM), 2 μl of cDNA, and dH_2_O to a final volume of 20 μl. The reaction mixtures were incubated in a 96-well plate at 95°C for 15 min, followed by 35 cycles of 94°C for 15 s, 60°C for 30 s, and 70°C for 30 s. All reactions were performed in triplicate. The primers for the microRNAs had the same sequences as the tick microRNAs with appropriate adjustments at their 5′ terminus. Mx3000/Mx Pro software (Stratagene, United States) was used to construct a melting curve. Standard curves with fivefold dilutions were performed for each assay, and PCR efficiency calculations were based on the slopes of the standard curves. The absolute amount of each microRNA was calculated using the 2^–△△*CT*^ method (Carbon et al., 2009) according to the standard curve. The housekeeping gene U6 was employed as an endogenous control (Duan et al., 2018), and the U6 primers were provided by QIAGEN Co. Ltd., China. Each sample was replicated three times. The microRNA level in various samples of developmental stages was determined individually. Each microRNA level is expressed as the 2^–△△*CT*^ mean ± SE. One-way ANOVA was applied to examine the significance of differential expression level in each mature/novel microRNA between eggs and larvae, larvae and adults, and eggs and adults, and the difference was considered significant at *P* < 0.05. Clones containing an insert of the correct size from four independent PCRs were sequenced on both strands using an ALF sequencer (Pharmacia Biotech).

**TABLE 1:** Alignment to GenBank and Rfam data libraries.

Category	Egg				Larvae				Nymph				Adult			
	Unique sRNAs	Percent (%)	Total sRNAs	Percent (%)	Unique sRNAs	Percent (%)	Total sRNAs	Percent (%)	Unique sRNAs	Percent (%)	Total sRNAs	Percent (%)	Unique sRNAs	Percent (%)	Total sRNAs	Percent (%)
Total	2,585,169	100	16,262,023	100	1,252,678	100	9,835,555	100	1,558,217	100	9,202,455	100	1,155,283	100	9,147,849	100
MiRNA	33,311	1.29	987,808	6.07	35,866	2.86	1,483,548	15.08	26,866	1.72	799,098	8.68	22,193	1.92	1,627,034	17.79
Rrna	41,612	1.61	1,322,480	8.13	58,098	4.64	602,546	6.13	33,881	2.17	772,142	8.39	23,876	2.07	1,023,248	11.19
Repeat	6	0.00	6	0.00	41	0.00	197	0.00	45	0.00	215	0.00	30	0.00	68	0.00
snRNA	2,245	0.09	12,537	0.08	1,862	0.15	8,234	0.08	920	0.06	2,527	0.03	448	0.04	1,134	0.01
snoRNA	104	0.00	134	0.00	166	0.01	253	0.00	87	0.01	120	0.00	989	0.09	1,428	0.02
Trna	11,969	0.46	247,535	1.52	19,779	1.58	183,847	1.87	16,681	1.07	174,613	1.90	5,462	0.47	49,742	0.54
Unann	2,495,922	96.55	13,691,523	84.19	1,136,866	90.75	7,556,930	76.83	1,479,737	94.96	7,453,740	81.00	1,102,285	95.41	6,445,195	70.46

*Annotated sRNA tags with rRNA, scRNA, snoRNA, snRNA, and tRNA from 13 GenBank libraries by BLAST. For some species, non-coding RNAs from GenBank need to be supplied as reference for this analysis.*

### Sequence Alignment and Phylogenetic Analysis

The mirBase^5^ accession numbers for miR-1 and cluster members are shown in Table 4. Multiple sequence alignments were analyzed using Clustalx (1.81) software. A phylogenetic tree was constructed with the sequences obtained in this study and sequences of miR-1 precursor sequences from different species available in the miRBase data library using neighbor joining in the MEGA 7 software (Livak and Schmittgen, 2001).

### Cell Culture and Luciferase Assay

The 293T cell line used in this study was maintained in Dulbecco’s modified Eagle’s medium (DMEM) (Gibco, Waltham, United States) supplemented with 10% fetal bovine serum (Gibco), penicillin, and streptomycin in an incubator with 5% CO_2_ at 37°C. Predicted binding sites were cloned and inserted into the pmirGLO vector (Promega, Madison, United States). For reporter assays, 150 ng of pmirGLO reporter vector and 50 nM miR-1 mimic (RiboBio, Guangzhou, China) were cotransfected into 293T cells using Lipofectamine 2000. No-mimic-treatment cells were used as a blank control, and cells carrying the pmirGLO-Hsp vector alone were used as the negative control. Firefly and Renilla luciferase activities were measured 48 h post-transfection with a Dual-Luciferase Reporter Assay System (Promega). First, 100 μl of luciferase assay reagent II was added to each well; firefly luciferase activities were measured. Subsequently, 100 μl of Stop&Glo reagent was added, and Renilla luciferase activities were measured. Firefly luciferase in the pmirGLO vector was used for normalization of Renilla luciferase expression. Treatments were assessed in triplicate, and transfections were repeated three times. Firefly luciferase activities were divided by Renilla luciferase activities for each experiment, providing the ratio.

### Synthesis and Application of Antagomir

Antagomirs, microRNA-specific antisense oligonucleotides, were synthetized by Dharmacon^6^. The microRNA-1 antagomir (Ant1) is the reverse complement of mature microRNA-1, and chemical modification was performed as described in a previous study (Kumar et al., 2016) (5′-mC.^∗^.mG.^∗^.mC.mG.mC.mG. mC.mU.mA.mC.mU.mU.mC.mA.mG.mG.mU.mA.mC.mC.^∗^.m U.^∗^.mG.^∗^.mA.^∗^-Chl-3′). The “missense” (MsAnt) sequence (5′-mC.^∗^.mG.^∗^.mC.mU.mU.mU.mC.mG.mU.mG.mG.mU.mU.mC. mU.mG.mG.mU.mA.mC.^∗^.mC.^∗^.mU.^∗^.mU.^∗^-Chl-3′) was used as the negative control for the antagomir [“^∗^” is a phosphate backbone modification that was introduced to increase nuclease resistance and facilitate cellular uptake and bioavailability *in vivo*. “m” is a 2′-O-methyl (2′-OMe) modification, which reduces off-targeting. “Chl” is cholesterol, which can enhance gene silencing *in vivo*]. Non-injected ticks were used as a blank control group. To assess the specificity of the antagomir, we measured the levels of other microRNAs, such as microRNA-10. Antagomirs were microinjected into unfed adult female *Ha. anatolicum* at a dose of 400 μM in 0.5 μl. Every group consisted of 30 female ticks, and the groups were given a blood meal on a host at 24 h after microinjection.

### Statistical Analysis

All data were analyzed with GraphPad 5 using Student’s *t*-test. Probability values of less than 0.05 were considered significant, and results are shown as the mean ± SEM.

## Results

### Small RNA Library Construction and Solexa Sequencing

**FIGURE 1:**
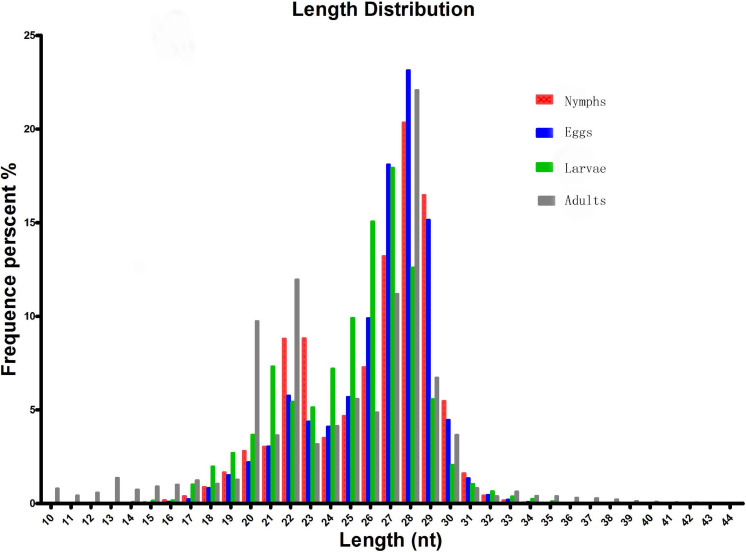
Length distribution and abundance of sequences in different developmental stages of *Hyalomma anatolicum anatolicum*. Contaminant reads from the fastq file were removed to obtain clean reads. Then, the length distribution of these clean reads was summarized. Normally, the length of small RNA is between 18 and 30 nt. The length distribution analysis is helpful to assess the compositions of small RNA samples. For example, miRNA is normally 21 or 22 nt, siRNA is 24 nt, and piRNA is 30 nt.

To identify microRNAs involved in different stages of *Ha. anatolicum* development, four sRNA libraries pooled from eggs, larvae, nymphs, and adults were constructed and sequenced using an Illumina HiSeq2000 high-throughput sequencer. The very basic figure from sequencing was converted into sequence data by the base calling step, and some contaminant reads from the fq file were removed to obtain the final clean reads; SOAP was used to map the sRNA tags to the genome. The program and parameters were as follows: SOAP −v 0 −r 2 −M 0 −a clean.fa -D ref_genome.fa.index -o match_genome.soap. Datasets from the four libraries were compared with the repository of mature animal microRNAs to known microRNAs in miRBase21^7^. As a result, a total of 16,262,023, 9,835,555, 9,202,455, and 9,147,849 raw reads were obtained for the egg, larval, nymph, and adult libraries, respectively. After the removal of low-quality reads, adaptors, and insufficient tags, 2,585,169, 1,252,678, 1,558,217, and 1,155,283 clean reads of 18–30 nt were obtained, respectively. Length distribution analysis showed that most reads were 21–30 nt long, with the highest percentage of reads (23.14%) being 28 nt long; 15.15% were 27 nt long (Figure 1).

**FIGURE 2:**
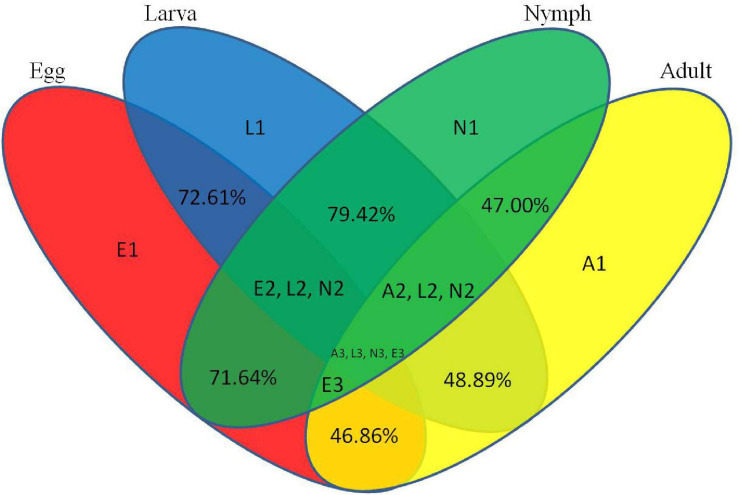
Common and specific unique tag sequences among samples. The percentages indicate common microRNAs among developmental stages. E1, L1, N1, and A1 indicate specific microRNAs in eggs, larvae, nymphs, and adults, respectively. “E2 L2 N2” indicates common microRNAs in eggs, larvae, and nymphs. “A2 L2 N2” indicates common microRNAs in adults, larvae, and nymphs. “E3” indicates common microRNAs in adults, eggs, nymphs, and adults. “A3 L3 N3 E3” indicates common microRNAs in all developmental stages.

To assess the efficiency of high-throughput sequencing for sRNA detection, all sequence reads from different development stages of *Ha. anatolicum* were annotated and classified through alignment with the GenBank and Rfam databases. These sequences accounted for 1.29, 2.86, 1.72, and 1.92% of unique sRNAs for eggs, larvae, nymphs, and adults, respectively. To confirm the known miRNAs, these sequences were perfectly mapped to the *Ixodes scapularis* reference genome (accession: PRJNA34667) (Table 1). In total, 72.61% sequences were common between larvae and eggs, 48.89% between larvae and adults, 46.86% between eggs and adults, and 47.00, 79.42, and 71.64% between nymphs and adults, nymphs and larvae, and nymphs and eggs, respectively, 9.67% miRNAs were common to larvae, nymphs, and adults (Figure 2).

### Known microRNAs and Differential Expression Analysis

**FIGURE 3:**
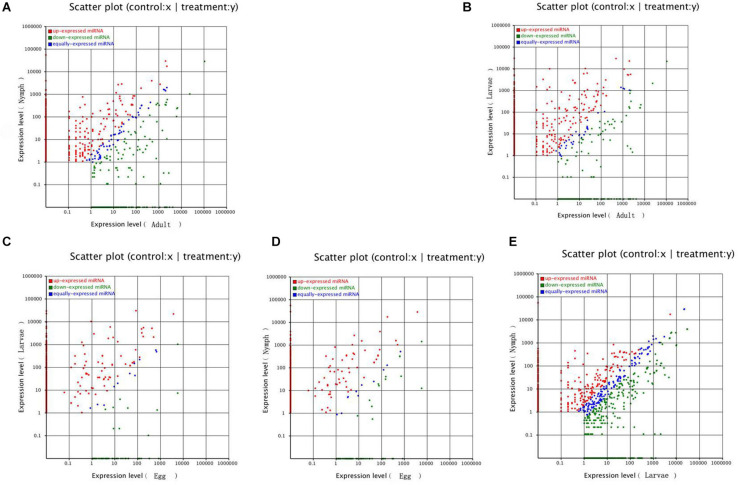
Comparison of differential expression levels of known microRNAs in different developmental stages. Each point in the figure represents a microRNA. The *X*-axis and *Y*-axis show the expression levels of microRNAs in the two samples. The red points represent microRNAs with ratios > 2; the blue points represent microRNAs with 1/2 < ratios ≤ 2; the green points represent microRNAs with ratios ≤ 1/2. Ratios = Normalized expression in the treatment/Normalized expression in the control. Panel **(A)** represents different expression between nymphs and adults; **(B)** represents different expression between larvae and adults; **(C)** represents different expression between larvae and eggs; **(D)** represents different expression between nymphs and eggs; **(E)** represents different expression between nymphs and larvae.

In this study, known microRNAs from *Ha. anatolicum* ticks were analyzed by miRBase21. The results showed a total of 232 known microRNAs in the egg stage, 1,051 in larvae, 1,122 in nymphs, and 743 in adults. In eggs, miR-4175-3p and miR-4419b were predominately expressed, with more than 100,000 reads, and certain microRNAs constituted 17.78% (260,875/1,467,411) of the total sequencing reads, suggesting that they are abundantly expressed during this period. The sequencing frequencies of 916 microRNAs were much lower than the 10 reads in larvae, but miR-184, miR-1, and miR-184b were predominately expressed. This was also observed at the nymph tick stage. However, in adults, miR-1-3p, miR-1, let-7-5p, miR-4486, and miR-84a were the most abundant, each with more than 100,000 reads. A total of 1,368 microRNAs displayed the lowest sequencing frequencies, with no more than 10 reads in larvae, nymphs, and adults. In the four libraries, miR-1-3p, miR-1, and miR-4175-3p were detected with high abundance (Additional file 1). Compared with microRNA expression in various developmental stages, 987 microRNAs were significantly differentially expressed, with a *P* < 0.01. When larvae were used as controls, 193 microRNAs were significantly differentially expressed in adults and 88 in eggs. Similarly, in nymphs, 355 microRNAs were differentially expressed. When using eggs as a control, 55 significantly differentially expressed microRNAs were detected in adult ticks and 74 in nymphs. When adults were used as a control, there were 222 significantly differentially expressed microRNAs in nymphs (Figure 3 and Additional File 2). These microRNAs were mainly expressed at low levels in different developmental stages, such as miR-12-5p, miR-1357, miR-1193-5p, bantam-b, and miR-252b.

Currently, there are 49 known microRNAs in *I. scapularis*, a species belonging to the prostriate hard-tick lineage (Krützfeldt et al., 2005; Griffiths-Jones et al., 2008), but there are no known microRNAs identified for *Ha. anatolicum* or other metastriate hard-tick species. Our results indicated no known microRNAs in the egg stage but 46 in the larval stage, 45 in the nymph stage, and 41 in the adult stage (Table 2).

**TABLE 2:** Known miRNA alignment and expression profile.

	miRNA	MiRNA*	miRNA-5P	miRNA-3P	miRNA precursors	Unique sRNAs matched to miRNA precursors	Total sRNAs matched to miRNA precursors
**Known miRNA in miRBase**	49	0	49	–	–		
Egg	0	0	0	0	0	0	0
Larvae	46	0	0	0	46	752	583,349
nymph	45	0	0	0	45	582	482,204
Adult	41	0	41	524	1,219,591		

Alignment of sRNA to the miRNA precursor of corresponding species (if there is no precursor information of that species in miRBase, mature miRNA is acceptable) to obtain the miRNA count. If there is miRNA information of the species in miRBase, sRNA tags are aligned to the miRNA precursor/mature miRNA of corresponding species in miRBase; if not, sRNA tags are aligned to the miRNA precursor/mature miRNA of all plants/animals in miRBase (see Additional File 1). Here, the miRNA* cannot be detected as it is metabolized by the Dicer enzyme.

### Identification of Novel microRNA Candidates

In addition to profiling known microRNAs, high-throughput sequencing has the advantage of revealing functionally important novel microRNAs that might not be detected using traditional methods. The unannotated unique sRNAs and total sRNAs are shown in Table 1. To determine whether these sRNA sequences are genuine tick microRNAs, 17, 30, 16, and 10 potential novel microRNAs in eggs, larvae, nymphs, and adults, respectively, were examined. The number of total novel microRNA candidates was 1,012, 4,143, 2,721, and 2,130, respectively, in these four developmental stages (Table 3). The characteristic hairpin structure of the microRNA precursor can be used to predict novel microRNAs. We used the prediction software Mireap to predict novel microRNAs by exploring the secondary structure, Dicer cleavage site, and minimum free energy of unannotated sRNA tags that could be mapped to the genome. Mireap can be accessed from the following link: http://sourceforge.net/projects/mireap/ (Zuker, 2003). The lengths ranged from 20 to 24 nt, with a typical stem-loop structure and free energy ranging from −43.5 to −19.4 kcal mol^–1^ (Additional File 3).

**TABLE 3:** Mapping the clean tags back to genome by SOAP2 to analyze the expression and distribution of sRNA tags across the genome.

Type	Unique sRNAs	Mapping to genome	Total sRNAs	Mapping to genome	Number of unique novel miRNA candidates	Number of total novel miRNA candidates
Eggs	2,585,169	79,076 (3.06%)	16,262,023	3,099,347 (19.06%)	17	1,012
Larvaes	1,252,678	29,211 (2.33%)	9,835,555	1,036,333 (10.54%)	30	4,143
Nymphs	1,558,217	20,176 (1.29%)	9,202,455	533,540 (5.80%)	16	2,721
Adults	1,155,283	15,034 (1.30%)	9,147,849	2,099,735 (22.95%)	10	2,130

*If the genome sequence of the species is not available, the corresponding EST or one specified species with available genome sequence as the reference can be analyzed. The characteristic hairpin structure of miRNA precursor can be used to predict novel miRNA. We developed Mireap (http://sourceforge.net/projects/mireap/), a prediction software to predict novel miRNA, by exploring the secondary structure, the Dicer cleavage site, and the minimum free energy of the unannotated sRNA tags that could be mapped to the genome. Li et al. (2009).*

### MicroRNA Target Gene Prediction and GO Enrichment

**FIGURE 4:**
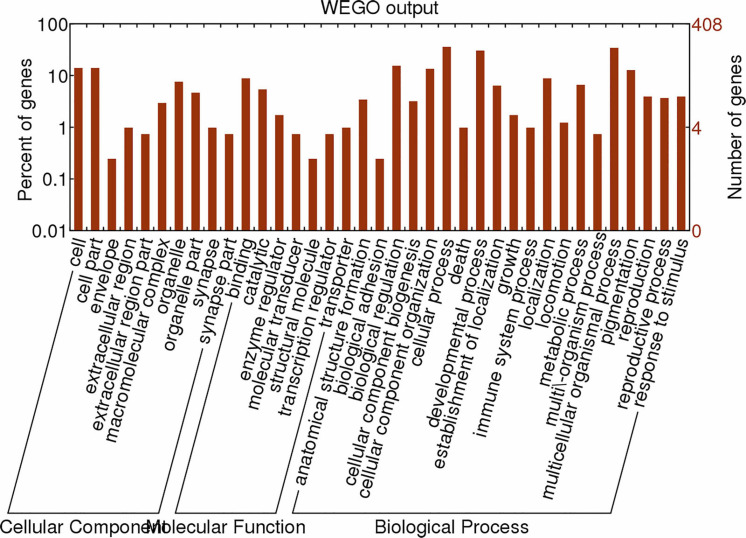
Partial GO classification annotated by Gene2GO for predicted target genes. The figure shows partial GO enrichment for the predicted target genes in terms of cellular components, molecular functions, and biological processes.

We screened microRNA 1 (miR-1), which exhibited a high expression level, as an important regulatory factor in ticks. To further understand the physiological functions and biological processes involving miR-1 during various developmental stages, target gene prediction was performed based on microRNA/mRNA interactions to provide molecular insight into processes. The MireapV0.2 software results revealed target genes involved tracheal system development, transcription factors, transmembrane receptors, and notum morphogenesis, among others (Additional File 4). Enrichment analysis was performed using GO^8^, an international standardized classification system for gene annotations that provides insight into the molecular functions of genes in various biological processes (Allen et al., 2005). In the cellular component category, there were 66 genes with a *P* ≤ 1. Moreover, 57.50% of the genes clustered into the term organelles. Regarding molecular function, 76 genes were assigned; most were related to catalytic activity, with 51 (67.80%) annotated genes. Analysis of biological processes showed that 266 genes are involved in macromolecule metabolic processes or nitrogen compound metabolic processes, at 36.80 and 23.20%, respectively. Figure 4 illustrates the global analysis of GO enrichment of targets.

### Analysis of miR-1 Conservation

**FIGURE 5:**
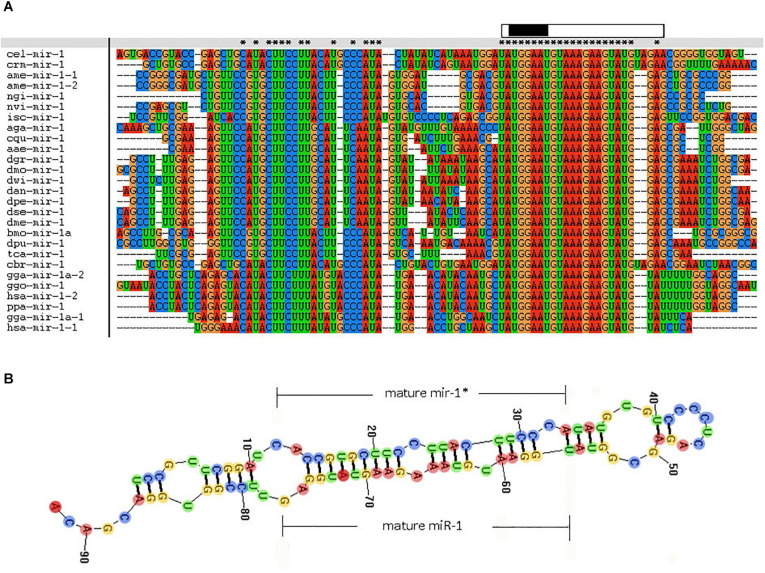
Comparative results of the nucleotide sequence of each species miR-1 (Clustal w software). “^∗^” indicates conserved sequences. “partial precursor sequence” is the precursor sequence of miR-1. “Mature” denotes the mature sequence of miR-1. “seed region” is the seed region of miR-1.

In addition, we examined miR-1 hairpin precursor sequences in different species. The 3′-arm of the hairpin is highly conserved, though the many changes in the 5′-arm are fully consistent with the precursor hairpin structure. Other miR-1 sequences are short and very similar, and their genomic contexts can improve our ability to annotate and explore their evolutionary origins (Figure 5A). The genomic organization of miR-1 cluster members across phyla suggests that miR-1 is an ancestral microRNA. The characteristic hairpin structure of microRNA precursors can be used to predict microRNAs. Mireap (Luo et al., 2019)^9^ was used to predict microRNA by exploring the secondary structure, Dicer cleavage site, and minimum free energy of the unannotated sRNA tags mapped to the genome (Figure 5B).

**FIGURE 6:**
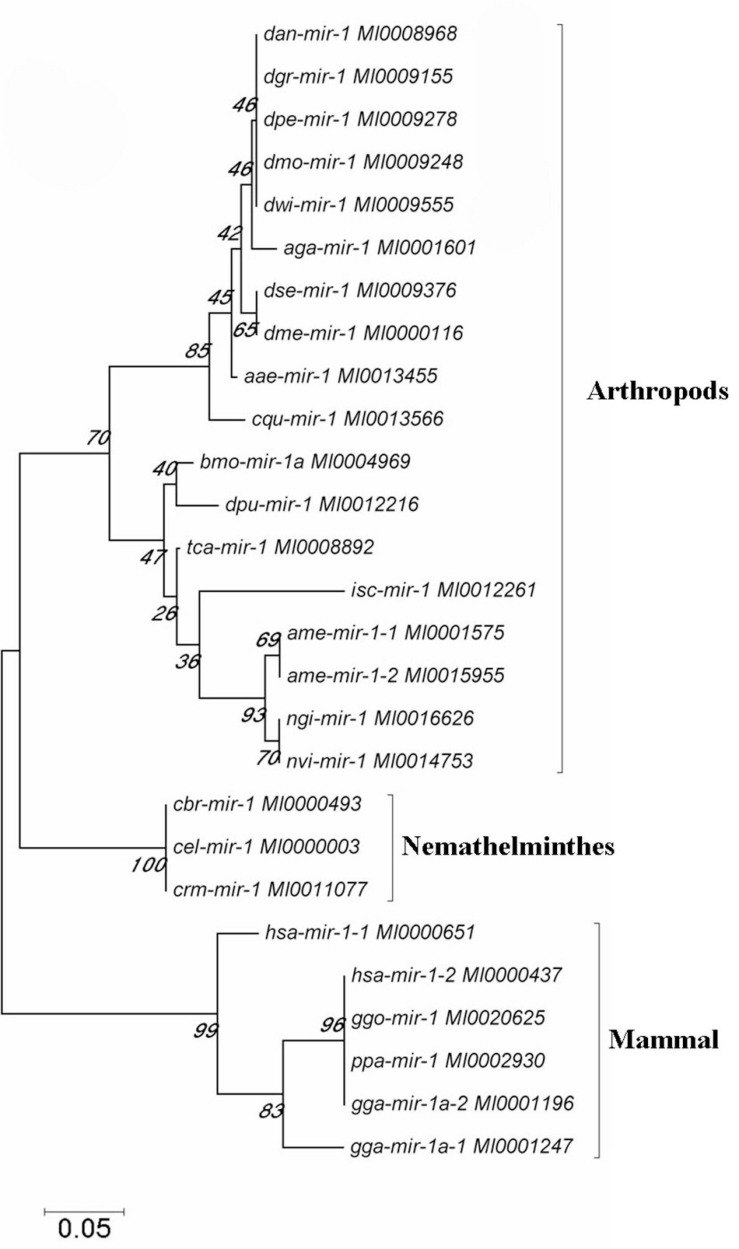
The phylogenetic tree was constructed for the miR-1 gene in selected species by neighbor-joining (MEGA 4.0 software) with maximum parsimony and 500 bootstrap replicates. Reference nucleotide sequences were selected by BLAST searches of the NCBI nt database.

We further investigated the evolutionary pattern of this gene in vertebrates and found that it has been generated by multiple replications of the ancestor gene, including two duplications as a whole and one fragmental replication, with mutation and deletion of certain genes in some species (Figures 5, 6). Analysis of the phylogenetic distribution of the miR-1 cluster in various species indicated that it is an ancient gene originating in the urochordate *Caenorhabditis elegans.* In contrast, *Drosophila melanogaster* contains only a single copy of the miR-1 gene, though the genomes of vertebrates contain more than one copy.

### miR-1 Targets Hsp60

**FIGURE 7:**
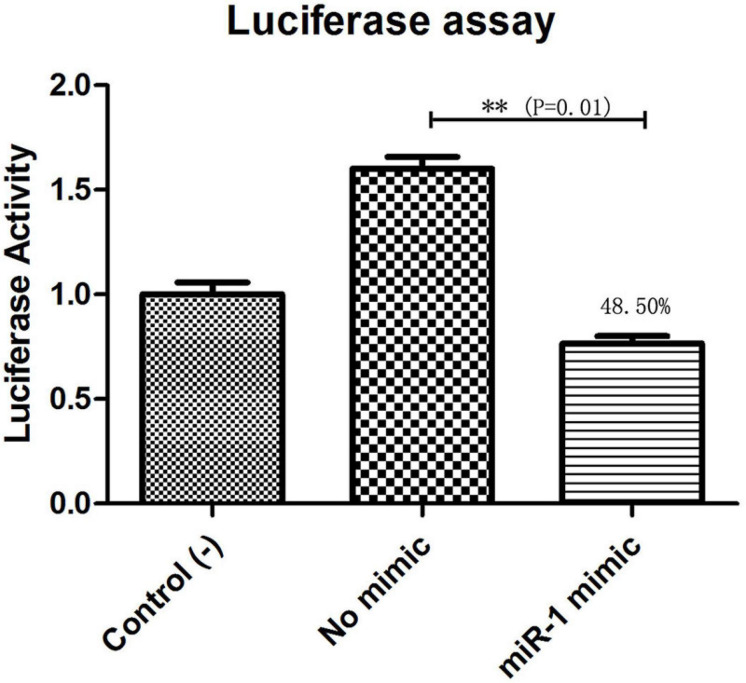
Hsp60 is a target of miR-1. Dual luciferase reporter assay results are represented as the mean ± SEM of triplicate samples. The recombinant plasmid pmirGLO containing the Hsp60 gene was transfected into 293T cells. Expression of Hsp60 was measured by qPCR. Data are presented as the mean ± SEM of triplicate samples. ^∗∗^*P* ≤ 0.01 (Student’s *t*-test).

The putative target gene (Hsp60) of miR-1 was identified using RNAhybrid, and the complete sequence of Hsp60 was used to predict binding sites. Computational analysis revealed many potential binding sites in Hsp60, but only one predicted result was within the 7-mer seed sequence sites (we chose the definition of the canonical seed binding site; the seed sequence is generally defined as 2–8 bases) in Hsp60 (Table 4). Based on these results, one binding site was cloned and inserted downstream of Renilla luciferase in the pmirGLO vector, which was then cotransfected into 293T cells with miR-1 mimics. According to luciferase reporter assays, only one site (position 342) resulted in 48.50% luciferase activity compared with that of the negative control and no-mimic control (Figure 7); the other site resulted in no significant difference compared with that of the control. Therefore, Hsp60 is a potential target of miR-1 *vitro*. Expression analysis of miR-1 and Hsp60 for various developmental stages and tissues in ticks was then performed.

**TABLE 4:** Putative miR-1 binding sites of HSP60.

miRNA	Position	mfe^*a*^	Target site
miR-1	342	−20.3kcal/mol	Target 5′ G CGCCCGCGAGG GAGC C A 3′ UCCAU GCUUU GCAU UCCA AGGUA UGAAG UGUA AGGU miRNA 3′ G AAA 5′

*^*a*^Minimum free energy (mfe) values based on RNAhybrid 2.2 prediction.*

To further confirm Hsp60 as an authentic miR-1 target gene *in vivo*, we conducted phenotype rescue experiments using Hsp60 RNAi in female ticks with an Ant-mir1 background; we speculated that RNAi-mediated knockdown of the physiologically relevant target of miR-1 would alleviate the adverse phenotypes caused by miR-1 depletion. Indeed, coinjection of Ant-mir1/dsRNA partially alleviated these phenotypes. Furthermore, the Ant-mir1/dsRNA female tick body weight significantly increased after a blood meal compared with that of dsHSP60 ticks. Thus, Hsp60 is an authentic target of miR-1 *in vivo*.

### Expression Analysis of miR-1 and Hsp60 for Various Developmental Stages and Tissues in Ticks

**FIGURE 8:**
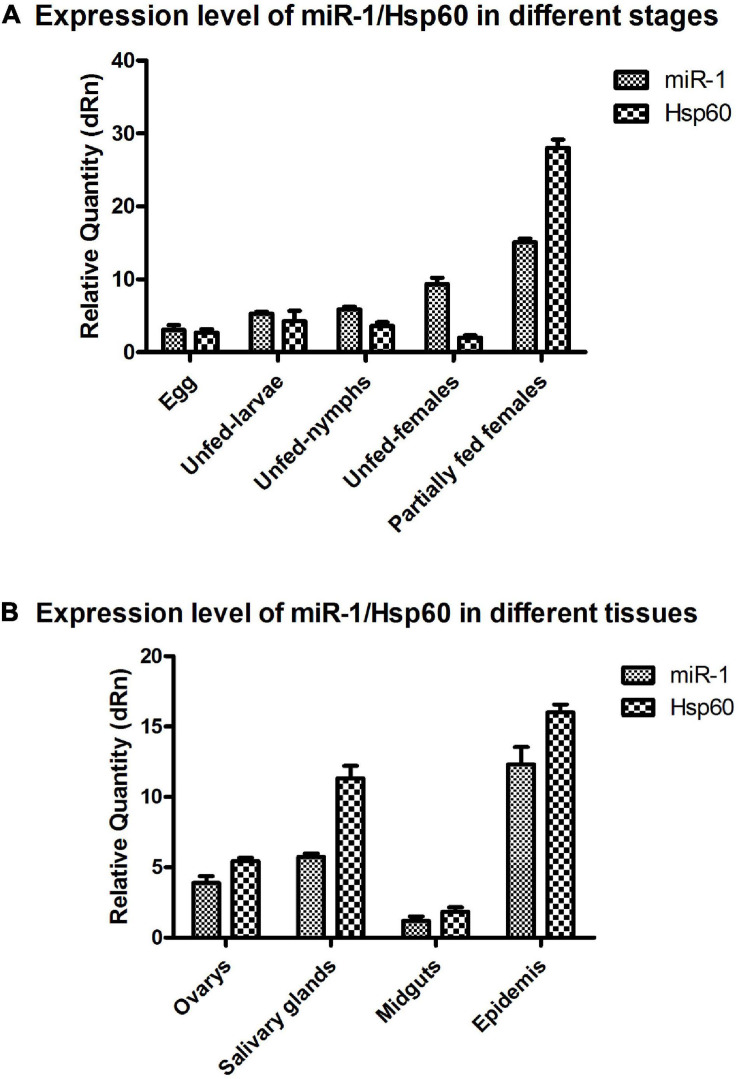
miR-1 and Hsp60 expression in different developmental stages and tissues. **(A)** Relative expression of Hsp60 and miR-1 was analyzed in eggs, unfed larvae, unfed nymphs, unfed adult females, and partially fed adult females. **(B)** Relative expression of Hsp60 and miR-1 in the ovary, salivary glands, midgut, and epidermis of fed adult female ticks. Data represent three biological replicates with three technical replicates and are shown as the mean ± SEM.

**FIGURE 9:**
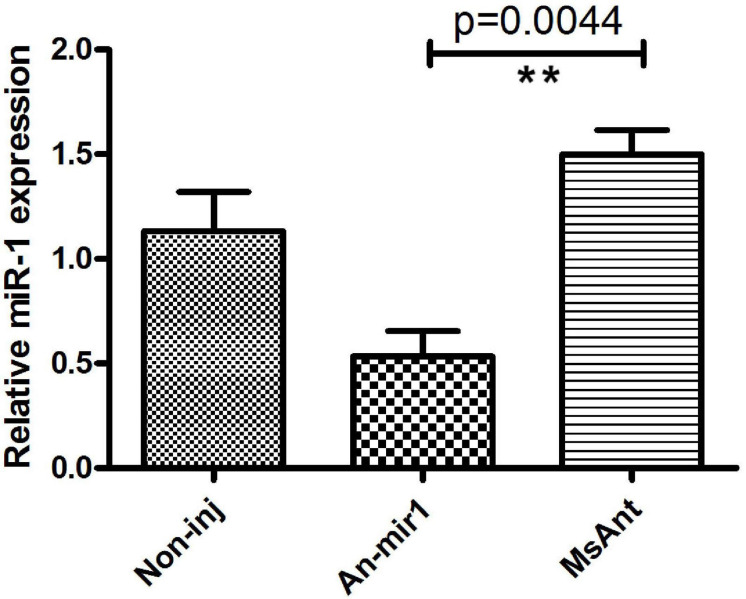
Relative mature miR-1 expression in fed female ticks treated with the miR-1 antagomir. Data represent three biological replicates with three technical replicates and are shown as the mean ± SEM. ^∗∗^*P* = 0.0044 < 0.01 (Student’s *t*-test).

**FIGURE 10:**
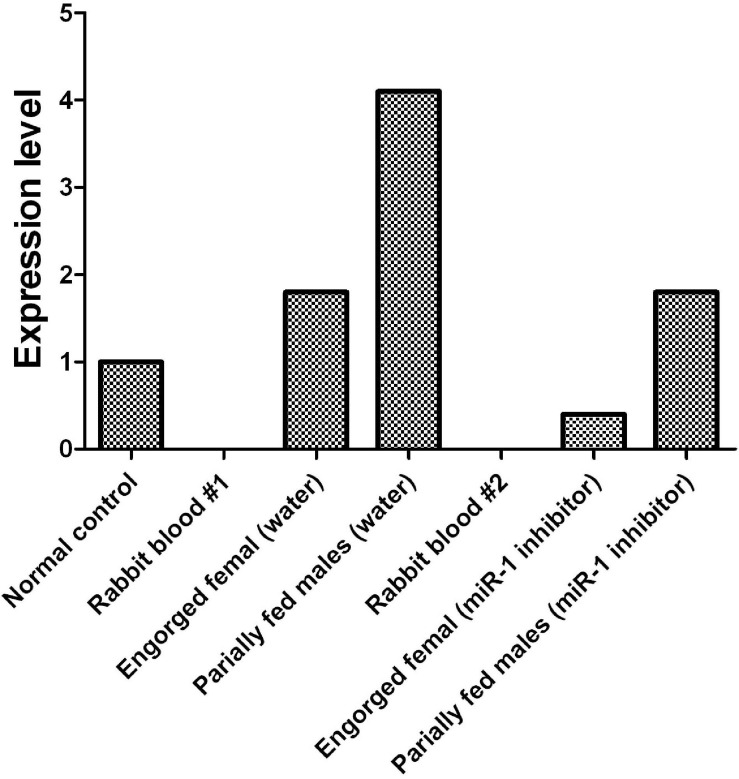
Before and after inhibition of miR-1, dynamic expression during tick engorgement was determined. Rabbit blood and water were used as controls.

To investigate the tissue- and developmental stage-specific expression of miR-1 in ticks, we measured expression levels of mature miR-1 at different developmental stages (egg, unfed larvae, fed larvae, unfed nymphs, fed nymphs, unfed adults, and fed adults) and in various tissues (the midgut, ovary, and salivary glands) of unfed and fed female ticks using real-time PCR. Expression analysis of mature miR-1 in different developmental stages showed that miR-1 expression peaked at the partially fed female stage (Figure 8A). In addition, expression of Hsp60 was highest at the engorged adult stage but was drastically reduced in eggs (Figure 8A). Analysis of different tissues indicated higher mature miR-1 levels in salivary glands than in other studied tissues in unfed female ticks; in fed female ticks, levels were highest in the epidermis (Figure 8B). To further investigate the potential function of miR-1 in adult female ticks, we silenced miR-1 by Ant-mir1: each unfed female tick was microinjected with Ant-mir1 or MsAnt, and after 48 h, real-time PCR was performed to assess the silencing efficiency of Ant-mir1. The results showed that miR-1 expression levels decreased to 64.67% after injection of Ant-mir1 compared with MsAnt and non-injection controls (Figure 9) (*t* = 5.800, df = 4). In this experiment, the host blood (rabbit) and male ticks were also examined by qPCR after injection of Ant-mir1, with insignificant levels in the rabbit blood (Figure 10).

### Physiological Effects of microRNA Inhibition on Ticks

Phenotypic manifestations were also monitored in female ticks after treatment with Ant-mir1, with a significant reduction in blood-feeding time after Ant-mir1 treatment compared with the MsAnt control group observed. According to analysis of the change in feeding, compared with the control groups, fed female ticks injected with Ant-mir1 showed a much faster increase in the duration of feeding for an average of 11 days. An average of 12 days after engorgement was found for the control group, and most female ticks began to lay eggs and lost weight. The weight of Ant-mir1-treated females was 81.00 ± 1.9 mg, but the MsAnt and non-injection groups weighed 127.20 ± 2.1 and 119.48 ± 2.0 mg, respectively, after engorgement, with average egg weights of 52.15 ± 3.2 and 48.96 ± 3.1, respectively. However, the spawning and mortality rates were not obviously affected (Table 5).

**TABLE 5:** Effect of vaccination with miR-1 inhibitor on tick feeding^*a*^.

Parameter	Immunized with		
	MsAnt control	Non-injection control	Injection Ant-1
Duration of feeding (days)	11–14	11–14	6–8**
Engorged weight, mean (mg)^*b*^	127.20 ± 2.1	119.48 ± 2.0	81.00 ± 1.9**
Spawning rate (%)	80.00	86.67	73.33
Average egg weight (mg)	52.15 ± 3.2	48.96 ± 3.1	48.51 ± 3.0
Mortality (%)^*c*^	20 ± 0.1	26 ± 0.6	23 ± 0.3

^*a*^Statistically significant (P < 0.05) as calculated by Student’s t-test. “**” Indicated **P = 0.0044 < 0.01 (Student’s t-test). ^*a*^Values are expressed as follows: average ± standard deviation. ^*b*^Dead ticks were excluded; calculated as batch average (batch weight/number of adult ticks). ^*c*^Mortality rate was calculated as the number of deaths during and after the feeding period.

### MicroRNA Analysis of the Hsp60 Gene

**FIGURE 11:**
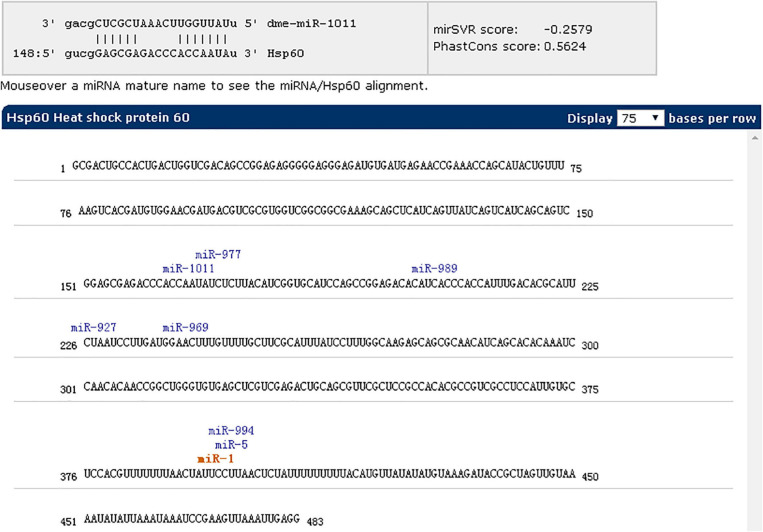
miR-1 inhibition as the model strain and Hsp60 as the target gene for microRNA cluster prediction using miRWalk (version 3.0).

**FIGURE 12:**
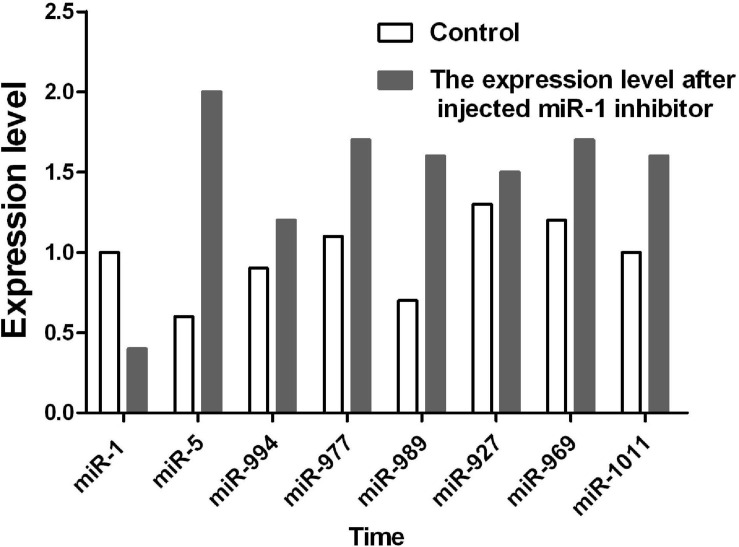
The expression level of the miR-1 cluster was detected by RT-PCR when miR-1 was inhibited.

After inhibition of miR-1, no significant changes in physiological indicators, including engorgement time, were detected. It may be that other members of the microRNA cluster compensate for the loss of miR-1. To confirm this finding, ticks with miR-1 inhibition were used as the model strain with Hsp60 as the target gene for microRNA cluster prediction. The results showed that other microRNAs were acting on the Hsp60 gene including mir-5, mir-994, and mir-969, among others (Figure 11). Therefore, RT-PCR was employed to detect expression levels of other microRNAs when miR-1 was inhibited; upregulation was found (Figure 12), with miR-5, which recognizes the same site as miR-1, being most significantly upregulated.

## Discussion

sRNAs are key molecules in organisms that induce gene silencing and have important roles in the regulation of cell growth, gene transcription, and translation. Small RNA digitalization analysis based on HiSeq high-throughput sequencing uses SBS sequencing, which can decrease loss of nucleotides caused by secondary structure. This approach is also beneficial because of the small sample quantity requirement, high throughput, and high accuracy via a simply operated automatic platform. Such analysis can result in millions of sRNA sequence tags in one run, comprehensively identify sRNAs of certain species under certain conditions, predict novel miRNAs, and assemble sRNA differential expression profiles between samples; overall, it is a powerful tool in sRNA functional research.

We analyzed sRNAs at different developmental stages in *Ha. anatolicum* by HiSeq sequencing. Assessment of fragment length to detect the peak of sRNA length distribution from ticks can help to determine relevant types of sRNA, such as miRNA of 28 to 22 nt, siRNA of 24 nt, and piRNA of 30 nt. In this study, 89% of the segments were in the range of 19∼24 nt (Figure 1), conforming to high-throughput sequencing quality control (Clop et al., 2006) and providing a foundation for the reliability of later relevant data analysis. The distribution of small molecular RNAs of different lengths during tick development may help to uncover important aspects of tick physiological functions (Wheeler et al., 2009). For example, miRNAs were the most abundant sRNA, with 1.29% unique to eggs, 2.86% unique to larvae, and 1.92% unique to adults. Thus, approximately 95% of the sRNA in different developmental stages is unannotated (Table 1), indicating a very complex regulatory system. As shown in Table 1, miRNAs composed a relatively large proportion of all the sRNAs annotated in larval ticks, indicating that miRNAs are involved in the regulation of gene expression in the early stage of tick development. This result can provide a basis for screening functional miRNAs by analyzing specific and common sequences as well as identifying targets for the study of specific regulatory molecules during tick development. Common miRNAs function in maintaining the physiological characteristics of ticks at different developmental stages (Figure 2). Nevertheless, specific expressed miRNAs may have important species-specific functions and/or host and pathogen specificity. These results provide a reliable basis for targeted study of miRNAs, and the differential miRNAs identified are of great utility for investigations of function.

Moreover, known miRNAs expressed in different samples were statistically analyzed to determine differences in expression level between two samples, and the differentially expressed miRNAs commonly expressed in the two samples were compared using a scatter plot (Log2 ratio) (Figure 3). Analysis of miRNA expression in different developmental stages showed approximately 800 miRNAs to be differentially expressed in these samples. For example, we detected 854 differentially expressed miRNAs between larvae and adults, 894 between nymphs and adults, 754 between eggs and nymphs, and 694 between eggs and larvae. In addition, using eggs as a control, Bantam and Let-7 miRNA and miR-133 showed large differences in various developmental stages. However, differential expression of miRNAs between adult and nymph and larval ticks was mainly found in the Let-7 family, such as Let-7 g, Let-7i, miR-144, and miR-1495. These results illustrate that different types of miRNA are expressed among different stages of development in the same tick species. It is notable that miR-1 is present not only at different developmental stages of ticks but also in abundance (millions of transcripts). This condition indicates that miR-1 plays an important physiological function in maintaining normal physiological metabolism in ticks. Previous reports have confirmed that miR-1 is involved in many biological processes in animals. In mammals, miR-1 regulates the development of myocardial cells, and abnormal regulation leads to heart diseases such as myocardial infarction, arrhythmia, and cardiac hypertrophy (Chen et al., 2011). Furthermore, the functions of miR-1 were assessed by GO analysis, revealing that miR-1 plays an important role in molecular binding and transcriptional regulation and is mainly involved in biological regulation, development, immune response, reproductive process, and cell death (Figure 4). Dysregulation of miR-1 may inhibit apoptosis or cause cell proliferation. Although multisequence alignment indicated significant differences in the nucleotide precursor sequences of miR-1 from different species, the mature sequence is conserved with regard to primary structure. Specifically, the “seed region” is identical among different species, though the “B region” and “C region” exhibit a difference of 2–3 bases (Figure 5). This difference occurs because the miRNA binds to target genes via the seed region, allowing specific binding between the miRNA and target gene; variable regions of mature sequences emphasize the diversity of these sequences. Such conservation in miRNAs has been applied for the early diagnosis of cancer, species identification, and pathogen detection as well as other purposes. The results also suggest both specificity and diversity in target mRNAs. In addition, we found significant differences in nucleic acid sequences for precursors of miR-1 and related species. In addition, these species clustered into three main branches in phylogenetic trees (Figure 6), demonstrating large differences among arthropods, Nemathelminthes, and primates. For example, ticks, mosquitoes, and *Drosophila* display high identity in the arthropod group. In particular, only the “C” region was identical, with 1–2 base differences in mature sequences of miR-1 from different species; the “seed region” was also identical. Therefore, miR-1 is an ancient regulatory molecule that plays an important role in the evolution of species.

In this study, it was shown that miR-1 is expressed at various developmental stages in tick development and in different tissues, and miR-1 expression was upregulated with the growth and development of ticks. In particular, the expression level was significantly higher during engorgement than starvation (Figure 7), and miR-1 was mainly expressed in the epidermis and midgut (Figure 7). These results indicate that miR-1 not only maintains normal physiological function but also has a leading role in tick muscle development. It is possible that with the engorging process, the tick’s muscles gradually extend to accommodate the filling of the midgut. To confirm this, miR-1 was inhibited via injection of an inhibitor, and the change in physiological indices was significant (Table 5). As time is required for inhibitors to function in living animals, the expression level of miR-1 in ticks was tracked to determine the optimal feeding time after injection, and miR-1 expression was downregulated significantly after 16 h (Figure 8). Ticks were released on the surface of animals for a long time and then collected, and total RNA was extracted to detect miR-1 expression (Figure 9).

Previous studies have shown that heat shock proteins (Hsps) are directly related to the immune protection caused by the invasion of foreign agents in a host (Wang et al., 2012). A previous report showed that a 63-kD symbiotic protein was abundant in probacterial symbionts in specialized cells of aphid hemolymph called mycetes; the protein is highly homologous (88.7% similar) to GroEL, a member of the Hsp60 family in *Escherichia coli* (Khan et al., 2009), and plays a role in viral spread. Our GO functional enrichment of microRNA analysis indicated that Hsp60 is a key target gene of miR-1 (as shown in Table 4 and Figure 10). miR-1 regulates the transcription of Hsp60, participating in the formation of specific tissues and organs during development. In other studies, the Hsp60 gene was associated with the development of the hematopoietic system and hematopoietic stem or progenitor cell proliferation, differentiation, maturation, and tumorigenesis (Soltys and Gupta, 1996; Samali et al., 1999; Xanthoudakis et al., 1999; Stanley and Fenton, 2000; Beere, 2005; Landgraf et al., 2007; Cappello et al., 2008). Therefore, it will be helpful to explore the functions of miR-1 to understand the biological characteristics of Hsp60 and its expression (Figure 7).

miR-1 is a highly conserved microRNA molecule, and to avoid the influence of rabbit-derived miR-1 on our results, rabbit blood was tested for miR-1. As no miR-1 was found in rabbit blood as a control, rabbit-derived microRNA would not affect tick physiology. In fact, expression was significantly lower than that in the control group in the miR-1 inhibition analysis, and the inhibitory effect was significant. Thus, in ticks, miR-1 might regulate Hsp60 to resist damage by foreign agents. The findings also indicate that the binding of animal miRNA to target mRNA depends not only on the seed region but that the “B” and “C” regions determine the specificity for the interaction in different species.

Previous studies have confirmed that miRNAs can be specifically expressed in high abundance in animal reproductive tissues, such as miR-449a, miR-465c, miR-202, and miR-547 (Yu et al., 2005). In addition, miR-34, miR-469, miR-465, and miR-101 are differentially expressed during testicular development (Novotny et al., 2007). Therefore, miRNA plays an important role in spermatogenesis, fertilized egg development, and gametic differentiation (Ro et al., 2007; Tang et al., 2007). In this study, the expression level of miR-1 in male ticks was significantly higher than that in female ticks, suggesting that miR-1 is key in the maturation of sperm. This conclusion needs to be confirmed by further experiments, which may provide ideas for studies on tick sexual reproduction and parthenogenesis. Our miRNA inhibition experiments confirmed that after mating with female ticks injected with miR-1 inhibitor, the spawning rate of males dropped to 40%, and the incubation rate was only 10%. Further observation of the developmental morphological characteristics of hatchling ticks revealed severe aberrations in secondary development after miR-1 inhibition. Furthermore, physiological indices such as engorgement weight and time and mortality of female ticks were analyzed, and it was found that the engorgement time of ticks injected with miR-1 inhibitor was significantly shorter than that of the control group (time = 7 ± 1 d for the experimental group, time = 12 ± 2 d for the control group). Regardless, there was no significant difference in other indicators (as shown in Table 5). To analyze the reasons for the lack of difference in physiological changes, we predicted other miRNAs possibly regulating Hsp60 and found that miR-5, miR-994, miR-969, miR-927, miR-989, miR-977, miR-1011, and miR-1 are common families that regulate the function of Hsp60 (Figure 11). We speculate that when miR-1 expression is inhibited, these cluster members will compensate for the loss. To verify this, qPCR was applied to detect expression of other miRNA clusters after miR-1 inhibition, which showed that other miRNAs were significantly upregulated after miR-1 was significantly inhibited. In fact, the expression level of miR-5, which has the same binding site as miR-1, was four times the normal level. miR-989 was also significantly upregulated (Figure 12). These results indicate that miR-5 plays an equally important role as miR-989 in the regulation of Hsp60 by miR-1. When miR-1 was inhibited, the two miRNAs preferentially compensated for the loss of function.

In conclusion, miR-1, a small non-coding RNA present in lower to higher animals, is highly conserved and is expressed to varying degrees in different stages of tick development and among tissues, with relatively stable expression. miR-1 plays an important role in tick development. However, considering that multiple genes are regulated by the same factors, loss of miR-1 function is compensated for by other miRNAs from the same cluster. Nonetheless, this compensation is not complete. In the early stage of development, the damage caused by abnormal gene expression may be temporarily negligible, but this damage cannot be effectively compensated for in later stages of development. Therefore, aberrant expression of miR-1 in ticks seriously affects their fecundity, especially egg hatching. This also leads to deformities later in development.

## Data Availability Statement

The original contributions presented in the study are publicly available. This data can be found here: the Hyalomma Anatolicum Anatolicum sequencing data obtained in this study have been deposited in the NCBI (SPA) database and obtained the entry numbers (larvae accession no. SUB8953582, nymph accession no. SUB8953797, adult accession no. SUB8928808, and egg accession no. SUB8953978).

## Ethics Statement

The animal study was reviewed and approved by the Ethics Committee of Lanzhou Veterinary Research Institute, Chinese Academy of Agricultural Sciences (approval no. LVRIAEC 2011-006).

## Author Contributions

GL and JL designed the experiments. XQ, QR, and JL performed the experiments. JXL and HY analyzed the data. JL and XL wrote the manuscript. WL, GZ, YT, and RC collected experimental materials. All authors read and approved the final version of the manuscript.

## Conflict of Interest

The authors declare that the research was conducted in the absence of any commercial or financial relationships that could be construed as a potential conflict of interest.

## Footnotes

1 http://soap.genomics.org.cn/soapdenovo.html:1.05

2 http://bowtie-bio.sourceforge.net/manual.shtml

3 http://www.bioinf.seu.edu.cn/microRNA/

4 http://www.geneontology.org/

5 http://www.mirbase.org/

6 http://dharmacon.gelifesciences.com

7 http://www.mirbase.org/

8 http://geneontology.org/

9 http://sourceforge.net/projects/mireap/

## Supplementary Material

Additional File 1 Differential expression of known microRNAs among developmental stages. A is a pair of developmental stages in the differential expression analysis; B is the microRNA name; C and D are adult total reads; E and F are true expression levels of microRNA; G and H (^∗^-std) are normalized expression levels of microRNA in a developmental stage; I (fold-change (log2^∗^/^∗^)) is fold change of microRNAs in the pair of developmental stages, with negative numbers indicating downregulation and positive numbers upregulation; J is the *P*-value reflecting the significance of microRNA differential expression between developmental stages, whereby a smaller value indicates greater significance of the difference in microRNA expression between developmental stages and the last column (sig-label) “^∗∗^”: fold_change (log2) > 1 or fold_change (log2) < −1, and *P* < 0.01. “^∗^”: fold_change (log2) > 1 or fold_change (log2) < −1, and 0.01 ≤ *P* < 0.05. None: Others.

Additional File 2 When no microRNA information of the species was available in miRBase21, small RNA tags were aligned to the microRNA precursor/mature microRNA of all plants/animals. The sequence and count of microRNA families (no specific species) found in the samples. A is the microRNA name (no specific species); B is the count of microRNAs of a cluster in the developmental stage; C is the sequence of a microRNA with the highest count in the cluster.

Additional File 3 The long sequence indicates microRNA precursor information in the order of sequence, name and length. The parentheses indicate microRNA precursor information in the order of hairpin structure, structure and MFE. “^∗∗∗^” and a short sequence indicate mature microRNA information in the order of sequence, name and length; “…” and a short sequence indicate information of matched sRNA tags in the order of sequence, ID, length, count.
