# Supplementary figures and images for: MicroRNA-1 Expression and Function in Hyalomma Anatolicum anatolicum (Acari: Ixodidae) Ticks
Source: Front Physiol. 2021 Apr 8;12:596289. doi: 10.3389/fphys.2021.596289 (PMC8061306; doi:10.3389/fphys.2021.596289)

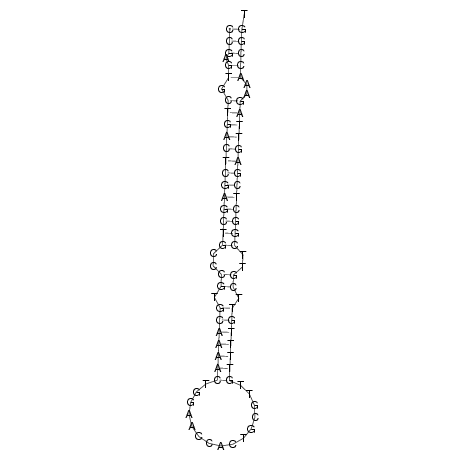

Supplement: Additional File 1 — Differential expression of known microRNAs among developmental stages. A is a pair of developmental stages in the differential expression analysis; B is the microRNA name; C and D are adult total reads; E and F are true expression levels of microRNA; G and H (∗-std) are normalized expression levels of microRNA in a developmental stage; I (fold-change (log2∗/∗)) is fold change of microRNAs in the pair of developmental stages, with negative numbers indicating downregulation and positive numbers upregulation; J is the P-value reflecting the significance of microRNA differential expression between developmental stages, whereby a smaller value indicates greater significance of the difference in microRNA expression between developmental stages and the last column (sig-label) “∗∗”: fold_change (log2) > 1 or fold_change (log2) < −1, and P < 0.01. “∗”: fold_change (log2) > 1 or fold_change (log2) < −1, and 0.01 ≤ P < 0.05. None: Others. [file Data_Sheet_1.ZIP › Addition 3-Adult/Adult-m0001.png]

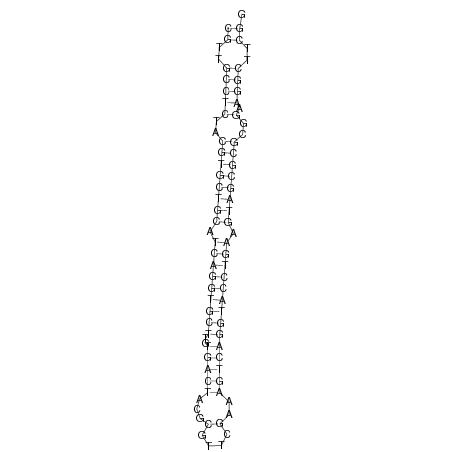

Supplement: Additional File 1 — Differential expression of known microRNAs among developmental stages. A is a pair of developmental stages in the differential expression analysis; B is the microRNA name; C and D are adult total reads; E and F are true expression levels of microRNA; G and H (∗-std) are normalized expression levels of microRNA in a developmental stage; I (fold-change (log2∗/∗)) is fold change of microRNAs in the pair of developmental stages, with negative numbers indicating downregulation and positive numbers upregulation; J is the P-value reflecting the significance of microRNA differential expression between developmental stages, whereby a smaller value indicates greater significance of the difference in microRNA expression between developmental stages and the last column (sig-label) “∗∗”: fold_change (log2) > 1 or fold_change (log2) < −1, and P < 0.01. “∗”: fold_change (log2) > 1 or fold_change (log2) < −1, and 0.01 ≤ P < 0.05. None: Others. [file Data_Sheet_1.ZIP › Addition 3-Adult/Adult-m0002.png]

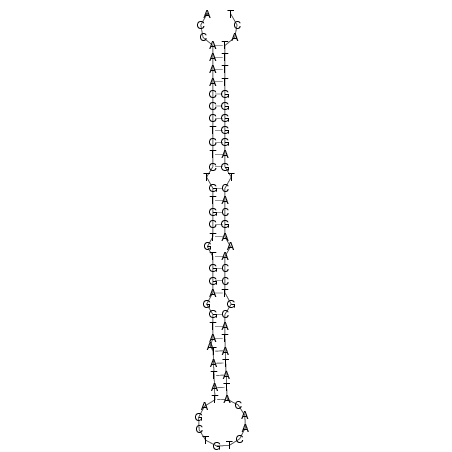

Supplement: Additional File 1 — Differential expression of known microRNAs among developmental stages. A is a pair of developmental stages in the differential expression analysis; B is the microRNA name; C and D are adult total reads; E and F are true expression levels of microRNA; G and H (∗-std) are normalized expression levels of microRNA in a developmental stage; I (fold-change (log2∗/∗)) is fold change of microRNAs in the pair of developmental stages, with negative numbers indicating downregulation and positive numbers upregulation; J is the P-value reflecting the significance of microRNA differential expression between developmental stages, whereby a smaller value indicates greater significance of the difference in microRNA expression between developmental stages and the last column (sig-label) “∗∗”: fold_change (log2) > 1 or fold_change (log2) < −1, and P < 0.01. “∗”: fold_change (log2) > 1 or fold_change (log2) < −1, and 0.01 ≤ P < 0.05. None: Others. [file Data_Sheet_1.ZIP › Addition 3-Adult/Adult-m0003.png]

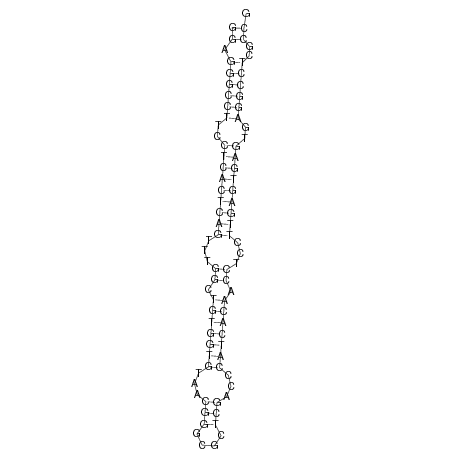

Supplement: Additional File 1 — Differential expression of known microRNAs among developmental stages. A is a pair of developmental stages in the differential expression analysis; B is the microRNA name; C and D are adult total reads; E and F are true expression levels of microRNA; G and H (∗-std) are normalized expression levels of microRNA in a developmental stage; I (fold-change (log2∗/∗)) is fold change of microRNAs in the pair of developmental stages, with negative numbers indicating downregulation and positive numbers upregulation; J is the P-value reflecting the significance of microRNA differential expression between developmental stages, whereby a smaller value indicates greater significance of the difference in microRNA expression between developmental stages and the last column (sig-label) “∗∗”: fold_change (log2) > 1 or fold_change (log2) < −1, and P < 0.01. “∗”: fold_change (log2) > 1 or fold_change (log2) < −1, and 0.01 ≤ P < 0.05. None: Others. [file Data_Sheet_1.ZIP › Addition 3-Adult/Adult-m0004.png]

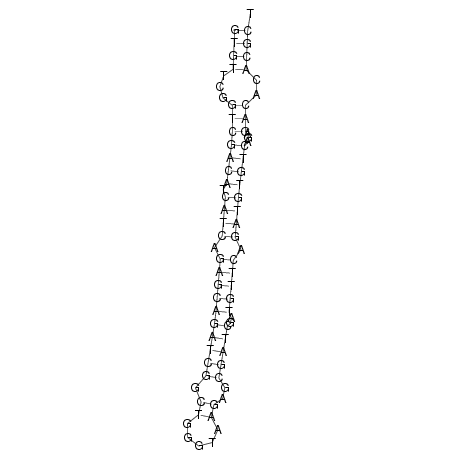

Supplement: Additional File 1 — Differential expression of known microRNAs among developmental stages. A is a pair of developmental stages in the differential expression analysis; B is the microRNA name; C and D are adult total reads; E and F are true expression levels of microRNA; G and H (∗-std) are normalized expression levels of microRNA in a developmental stage; I (fold-change (log2∗/∗)) is fold change of microRNAs in the pair of developmental stages, with negative numbers indicating downregulation and positive numbers upregulation; J is the P-value reflecting the significance of microRNA differential expression between developmental stages, whereby a smaller value indicates greater significance of the difference in microRNA expression between developmental stages and the last column (sig-label) “∗∗”: fold_change (log2) > 1 or fold_change (log2) < −1, and P < 0.01. “∗”: fold_change (log2) > 1 or fold_change (log2) < −1, and 0.01 ≤ P < 0.05. None: Others. [file Data_Sheet_1.ZIP › Addition 3-Adult/Adult-m0005.png]

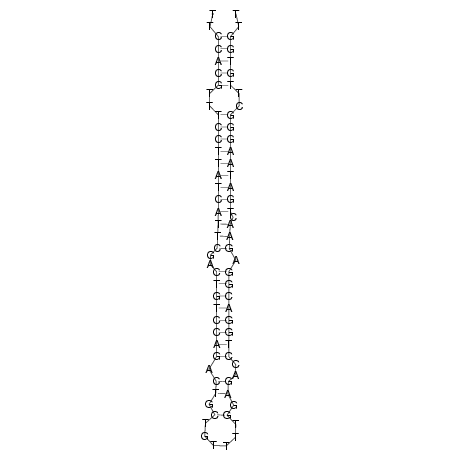

Supplement: Additional File 1 — Differential expression of known microRNAs among developmental stages. A is a pair of developmental stages in the differential expression analysis; B is the microRNA name; C and D are adult total reads; E and F are true expression levels of microRNA; G and H (∗-std) are normalized expression levels of microRNA in a developmental stage; I (fold-change (log2∗/∗)) is fold change of microRNAs in the pair of developmental stages, with negative numbers indicating downregulation and positive numbers upregulation; J is the P-value reflecting the significance of microRNA differential expression between developmental stages, whereby a smaller value indicates greater significance of the difference in microRNA expression between developmental stages and the last column (sig-label) “∗∗”: fold_change (log2) > 1 or fold_change (log2) < −1, and P < 0.01. “∗”: fold_change (log2) > 1 or fold_change (log2) < −1, and 0.01 ≤ P < 0.05. None: Others. [file Data_Sheet_1.ZIP › Addition 3-Adult/Adult-m0006.png]

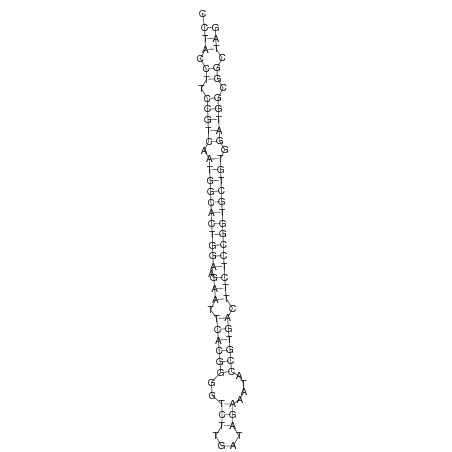

Supplement: Additional File 1 — Differential expression of known microRNAs among developmental stages. A is a pair of developmental stages in the differential expression analysis; B is the microRNA name; C and D are adult total reads; E and F are true expression levels of microRNA; G and H (∗-std) are normalized expression levels of microRNA in a developmental stage; I (fold-change (log2∗/∗)) is fold change of microRNAs in the pair of developmental stages, with negative numbers indicating downregulation and positive numbers upregulation; J is the P-value reflecting the significance of microRNA differential expression between developmental stages, whereby a smaller value indicates greater significance of the difference in microRNA expression between developmental stages and the last column (sig-label) “∗∗”: fold_change (log2) > 1 or fold_change (log2) < −1, and P < 0.01. “∗”: fold_change (log2) > 1 or fold_change (log2) < −1, and 0.01 ≤ P < 0.05. None: Others. [file Data_Sheet_1.ZIP › Addition 3-Adult/Adult-m0007.png]

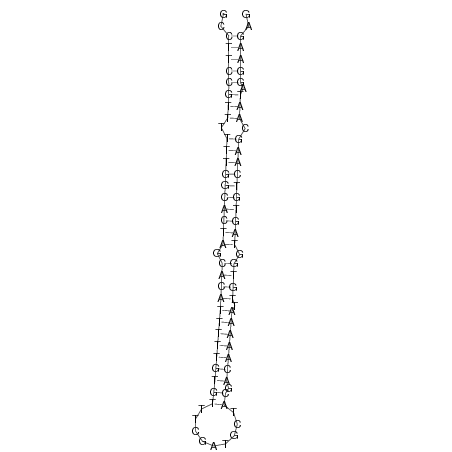

Supplement: Additional File 1 — Differential expression of known microRNAs among developmental stages. A is a pair of developmental stages in the differential expression analysis; B is the microRNA name; C and D are adult total reads; E and F are true expression levels of microRNA; G and H (∗-std) are normalized expression levels of microRNA in a developmental stage; I (fold-change (log2∗/∗)) is fold change of microRNAs in the pair of developmental stages, with negative numbers indicating downregulation and positive numbers upregulation; J is the P-value reflecting the significance of microRNA differential expression between developmental stages, whereby a smaller value indicates greater significance of the difference in microRNA expression between developmental stages and the last column (sig-label) “∗∗”: fold_change (log2) > 1 or fold_change (log2) < −1, and P < 0.01. “∗”: fold_change (log2) > 1 or fold_change (log2) < −1, and 0.01 ≤ P < 0.05. None: Others. [file Data_Sheet_1.ZIP › Addition 3-Adult/Adult-m0008.png]

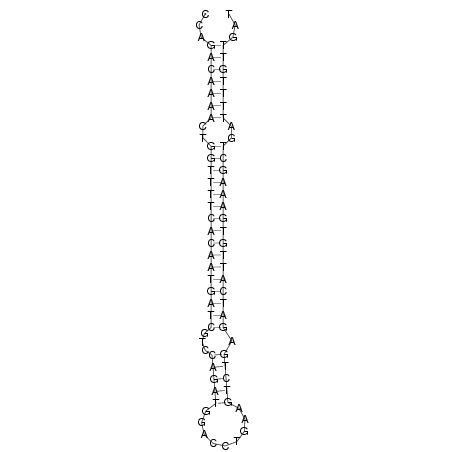

Supplement: Additional File 1 — Differential expression of known microRNAs among developmental stages. A is a pair of developmental stages in the differential expression analysis; B is the microRNA name; C and D are adult total reads; E and F are true expression levels of microRNA; G and H (∗-std) are normalized expression levels of microRNA in a developmental stage; I (fold-change (log2∗/∗)) is fold change of microRNAs in the pair of developmental stages, with negative numbers indicating downregulation and positive numbers upregulation; J is the P-value reflecting the significance of microRNA differential expression between developmental stages, whereby a smaller value indicates greater significance of the difference in microRNA expression between developmental stages and the last column (sig-label) “∗∗”: fold_change (log2) > 1 or fold_change (log2) < −1, and P < 0.01. “∗”: fold_change (log2) > 1 or fold_change (log2) < −1, and 0.01 ≤ P < 0.05. None: Others. [file Data_Sheet_1.ZIP › Addition 3-Adult/Adult-m0009.png]

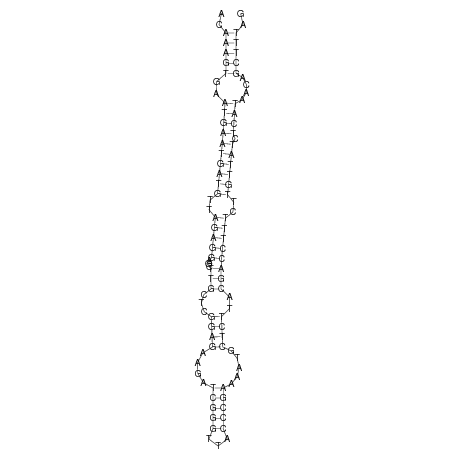

Supplement: Additional File 1 — Differential expression of known microRNAs among developmental stages. A is a pair of developmental stages in the differential expression analysis; B is the microRNA name; C and D are adult total reads; E and F are true expression levels of microRNA; G and H (∗-std) are normalized expression levels of microRNA in a developmental stage; I (fold-change (log2∗/∗)) is fold change of microRNAs in the pair of developmental stages, with negative numbers indicating downregulation and positive numbers upregulation; J is the P-value reflecting the significance of microRNA differential expression between developmental stages, whereby a smaller value indicates greater significance of the difference in microRNA expression between developmental stages and the last column (sig-label) “∗∗”: fold_change (log2) > 1 or fold_change (log2) < −1, and P < 0.01. “∗”: fold_change (log2) > 1 or fold_change (log2) < −1, and 0.01 ≤ P < 0.05. None: Others. [file Data_Sheet_1.ZIP › Addition 3-Egg/Egg-m0001.png]

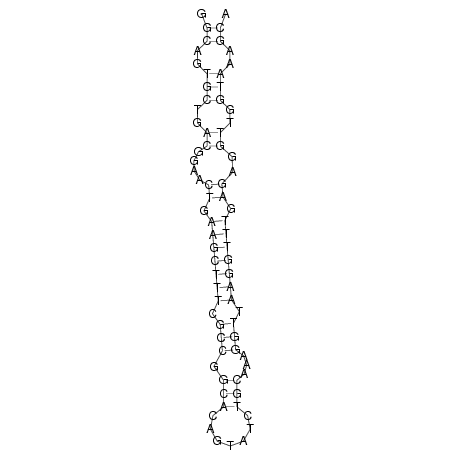

Supplement: Additional File 1 — Differential expression of known microRNAs among developmental stages. A is a pair of developmental stages in the differential expression analysis; B is the microRNA name; C and D are adult total reads; E and F are true expression levels of microRNA; G and H (∗-std) are normalized expression levels of microRNA in a developmental stage; I (fold-change (log2∗/∗)) is fold change of microRNAs in the pair of developmental stages, with negative numbers indicating downregulation and positive numbers upregulation; J is the P-value reflecting the significance of microRNA differential expression between developmental stages, whereby a smaller value indicates greater significance of the difference in microRNA expression between developmental stages and the last column (sig-label) “∗∗”: fold_change (log2) > 1 or fold_change (log2) < −1, and P < 0.01. “∗”: fold_change (log2) > 1 or fold_change (log2) < −1, and 0.01 ≤ P < 0.05. None: Others. [file Data_Sheet_1.ZIP › Addition 3-Egg/Egg-m0002.png]

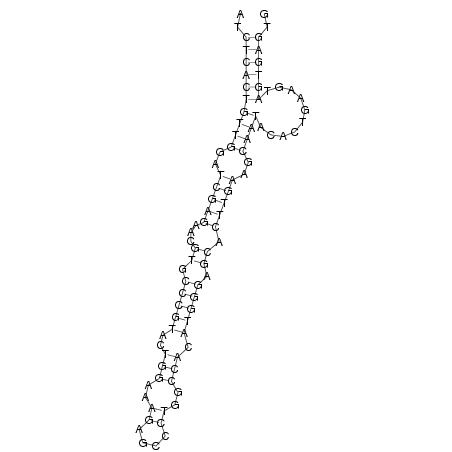

Supplement: Additional File 1 — Differential expression of known microRNAs among developmental stages. A is a pair of developmental stages in the differential expression analysis; B is the microRNA name; C and D are adult total reads; E and F are true expression levels of microRNA; G and H (∗-std) are normalized expression levels of microRNA in a developmental stage; I (fold-change (log2∗/∗)) is fold change of microRNAs in the pair of developmental stages, with negative numbers indicating downregulation and positive numbers upregulation; J is the P-value reflecting the significance of microRNA differential expression between developmental stages, whereby a smaller value indicates greater significance of the difference in microRNA expression between developmental stages and the last column (sig-label) “∗∗”: fold_change (log2) > 1 or fold_change (log2) < −1, and P < 0.01. “∗”: fold_change (log2) > 1 or fold_change (log2) < −1, and 0.01 ≤ P < 0.05. None: Others. [file Data_Sheet_1.ZIP › Addition 3-Egg/Egg-m0003.png]

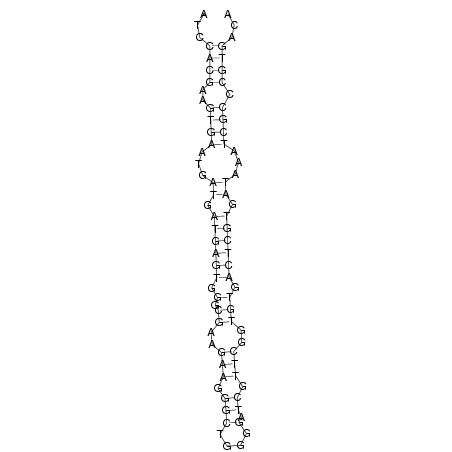

Supplement: Additional File 1 — Differential expression of known microRNAs among developmental stages. A is a pair of developmental stages in the differential expression analysis; B is the microRNA name; C and D are adult total reads; E and F are true expression levels of microRNA; G and H (∗-std) are normalized expression levels of microRNA in a developmental stage; I (fold-change (log2∗/∗)) is fold change of microRNAs in the pair of developmental stages, with negative numbers indicating downregulation and positive numbers upregulation; J is the P-value reflecting the significance of microRNA differential expression between developmental stages, whereby a smaller value indicates greater significance of the difference in microRNA expression between developmental stages and the last column (sig-label) “∗∗”: fold_change (log2) > 1 or fold_change (log2) < −1, and P < 0.01. “∗”: fold_change (log2) > 1 or fold_change (log2) < −1, and 0.01 ≤ P < 0.05. None: Others. [file Data_Sheet_1.ZIP › Addition 3-Egg/Egg-m0004.png]

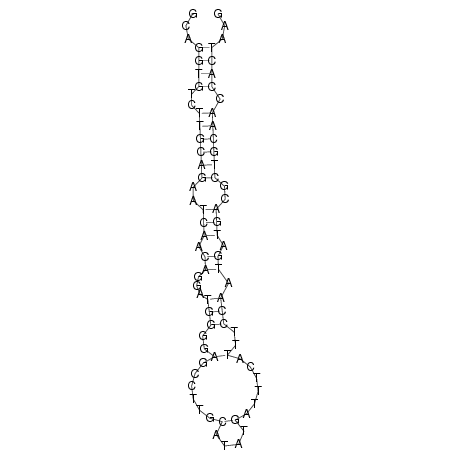

Supplement: Additional File 1 — Differential expression of known microRNAs among developmental stages. A is a pair of developmental stages in the differential expression analysis; B is the microRNA name; C and D are adult total reads; E and F are true expression levels of microRNA; G and H (∗-std) are normalized expression levels of microRNA in a developmental stage; I (fold-change (log2∗/∗)) is fold change of microRNAs in the pair of developmental stages, with negative numbers indicating downregulation and positive numbers upregulation; J is the P-value reflecting the significance of microRNA differential expression between developmental stages, whereby a smaller value indicates greater significance of the difference in microRNA expression between developmental stages and the last column (sig-label) “∗∗”: fold_change (log2) > 1 or fold_change (log2) < −1, and P < 0.01. “∗”: fold_change (log2) > 1 or fold_change (log2) < −1, and 0.01 ≤ P < 0.05. None: Others. [file Data_Sheet_1.ZIP › Addition 3-Egg/Egg-m0005.png]

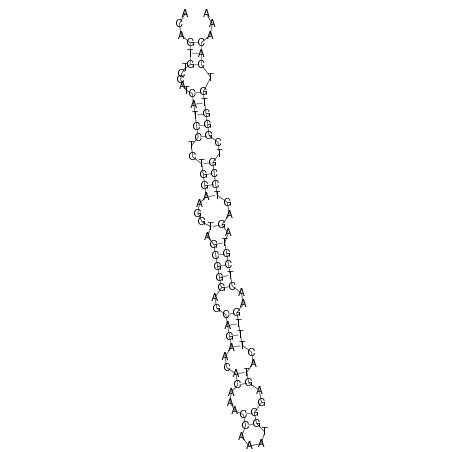

Supplement: Additional File 1 — Differential expression of known microRNAs among developmental stages. A is a pair of developmental stages in the differential expression analysis; B is the microRNA name; C and D are adult total reads; E and F are true expression levels of microRNA; G and H (∗-std) are normalized expression levels of microRNA in a developmental stage; I (fold-change (log2∗/∗)) is fold change of microRNAs in the pair of developmental stages, with negative numbers indicating downregulation and positive numbers upregulation; J is the P-value reflecting the significance of microRNA differential expression between developmental stages, whereby a smaller value indicates greater significance of the difference in microRNA expression between developmental stages and the last column (sig-label) “∗∗”: fold_change (log2) > 1 or fold_change (log2) < −1, and P < 0.01. “∗”: fold_change (log2) > 1 or fold_change (log2) < −1, and 0.01 ≤ P < 0.05. None: Others. [file Data_Sheet_1.ZIP › Addition 3-Egg/Egg-m0006.png]

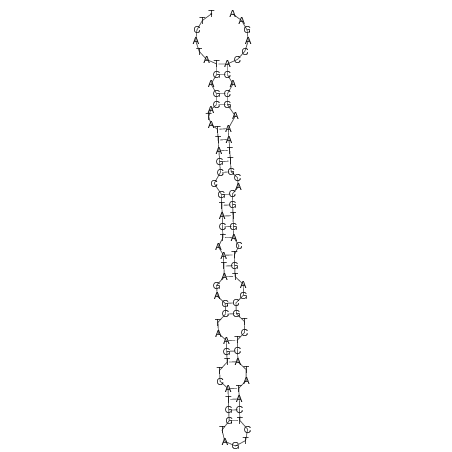

Supplement: Additional File 1 — Differential expression of known microRNAs among developmental stages. A is a pair of developmental stages in the differential expression analysis; B is the microRNA name; C and D are adult total reads; E and F are true expression levels of microRNA; G and H (∗-std) are normalized expression levels of microRNA in a developmental stage; I (fold-change (log2∗/∗)) is fold change of microRNAs in the pair of developmental stages, with negative numbers indicating downregulation and positive numbers upregulation; J is the P-value reflecting the significance of microRNA differential expression between developmental stages, whereby a smaller value indicates greater significance of the difference in microRNA expression between developmental stages and the last column (sig-label) “∗∗”: fold_change (log2) > 1 or fold_change (log2) < −1, and P < 0.01. “∗”: fold_change (log2) > 1 or fold_change (log2) < −1, and 0.01 ≤ P < 0.05. None: Others. [file Data_Sheet_1.ZIP › Addition 3-Egg/Egg-m0007.png]

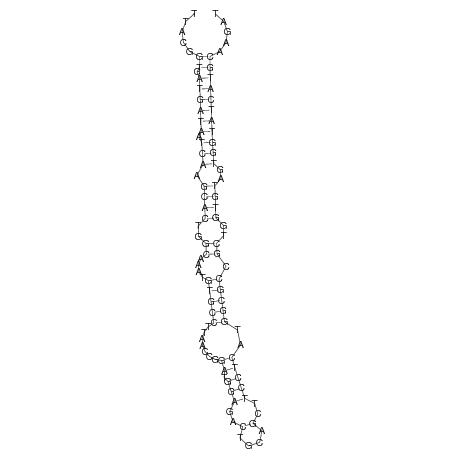

Supplement: Additional File 1 — Differential expression of known microRNAs among developmental stages. A is a pair of developmental stages in the differential expression analysis; B is the microRNA name; C and D are adult total reads; E and F are true expression levels of microRNA; G and H (∗-std) are normalized expression levels of microRNA in a developmental stage; I (fold-change (log2∗/∗)) is fold change of microRNAs in the pair of developmental stages, with negative numbers indicating downregulation and positive numbers upregulation; J is the P-value reflecting the significance of microRNA differential expression between developmental stages, whereby a smaller value indicates greater significance of the difference in microRNA expression between developmental stages and the last column (sig-label) “∗∗”: fold_change (log2) > 1 or fold_change (log2) < −1, and P < 0.01. “∗”: fold_change (log2) > 1 or fold_change (log2) < −1, and 0.01 ≤ P < 0.05. None: Others. [file Data_Sheet_1.ZIP › Addition 3-Egg/Egg-m0008.png]

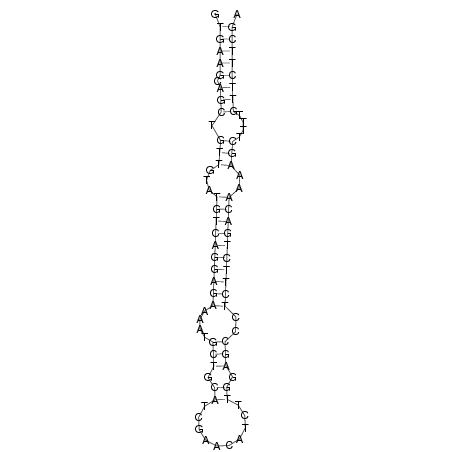

Supplement: Additional File 1 — Differential expression of known microRNAs among developmental stages. A is a pair of developmental stages in the differential expression analysis; B is the microRNA name; C and D are adult total reads; E and F are true expression levels of microRNA; G and H (∗-std) are normalized expression levels of microRNA in a developmental stage; I (fold-change (log2∗/∗)) is fold change of microRNAs in the pair of developmental stages, with negative numbers indicating downregulation and positive numbers upregulation; J is the P-value reflecting the significance of microRNA differential expression between developmental stages, whereby a smaller value indicates greater significance of the difference in microRNA expression between developmental stages and the last column (sig-label) “∗∗”: fold_change (log2) > 1 or fold_change (log2) < −1, and P < 0.01. “∗”: fold_change (log2) > 1 or fold_change (log2) < −1, and 0.01 ≤ P < 0.05. None: Others. [file Data_Sheet_1.ZIP › Addition 3-Egg/Egg-m0009.png]

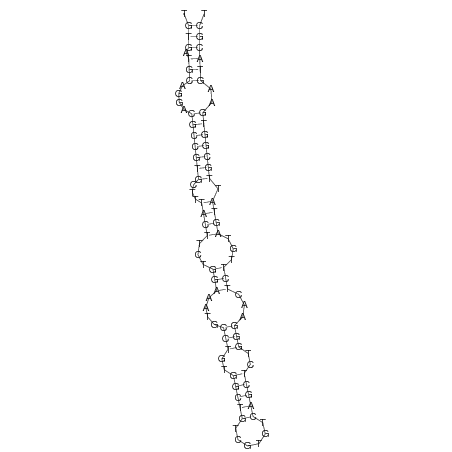

Supplement: Additional File 1 — Differential expression of known microRNAs among developmental stages. A is a pair of developmental stages in the differential expression analysis; B is the microRNA name; C and D are adult total reads; E and F are true expression levels of microRNA; G and H (∗-std) are normalized expression levels of microRNA in a developmental stage; I (fold-change (log2∗/∗)) is fold change of microRNAs in the pair of developmental stages, with negative numbers indicating downregulation and positive numbers upregulation; J is the P-value reflecting the significance of microRNA differential expression between developmental stages, whereby a smaller value indicates greater significance of the difference in microRNA expression between developmental stages and the last column (sig-label) “∗∗”: fold_change (log2) > 1 or fold_change (log2) < −1, and P < 0.01. “∗”: fold_change (log2) > 1 or fold_change (log2) < −1, and 0.01 ≤ P < 0.05. None: Others. [file Data_Sheet_1.ZIP › Addition 3-Egg/Egg-m0010.png]

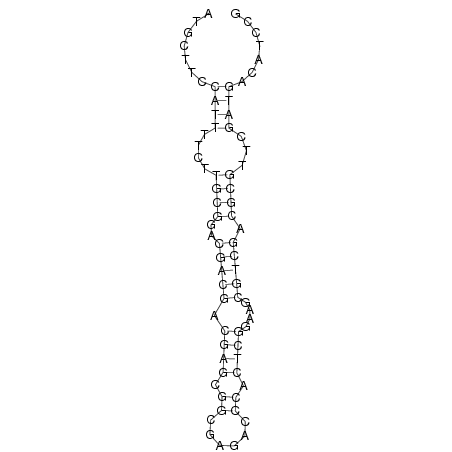

Supplement: Additional File 1 — Differential expression of known microRNAs among developmental stages. A is a pair of developmental stages in the differential expression analysis; B is the microRNA name; C and D are adult total reads; E and F are true expression levels of microRNA; G and H (∗-std) are normalized expression levels of microRNA in a developmental stage; I (fold-change (log2∗/∗)) is fold change of microRNAs in the pair of developmental stages, with negative numbers indicating downregulation and positive numbers upregulation; J is the P-value reflecting the significance of microRNA differential expression between developmental stages, whereby a smaller value indicates greater significance of the difference in microRNA expression between developmental stages and the last column (sig-label) “∗∗”: fold_change (log2) > 1 or fold_change (log2) < −1, and P < 0.01. “∗”: fold_change (log2) > 1 or fold_change (log2) < −1, and 0.01 ≤ P < 0.05. None: Others. [file Data_Sheet_1.ZIP › Addition 3-Egg/Egg-m0011.png]

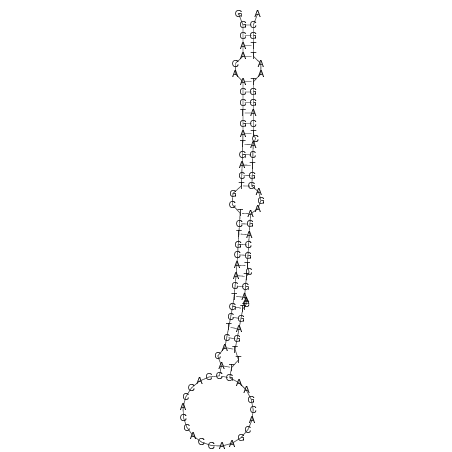

Supplement: Additional File 1 — Differential expression of known microRNAs among developmental stages. A is a pair of developmental stages in the differential expression analysis; B is the microRNA name; C and D are adult total reads; E and F are true expression levels of microRNA; G and H (∗-std) are normalized expression levels of microRNA in a developmental stage; I (fold-change (log2∗/∗)) is fold change of microRNAs in the pair of developmental stages, with negative numbers indicating downregulation and positive numbers upregulation; J is the P-value reflecting the significance of microRNA differential expression between developmental stages, whereby a smaller value indicates greater significance of the difference in microRNA expression between developmental stages and the last column (sig-label) “∗∗”: fold_change (log2) > 1 or fold_change (log2) < −1, and P < 0.01. “∗”: fold_change (log2) > 1 or fold_change (log2) < −1, and 0.01 ≤ P < 0.05. None: Others. [file Data_Sheet_1.ZIP › Addition 3-Egg/Egg-m0012.png]

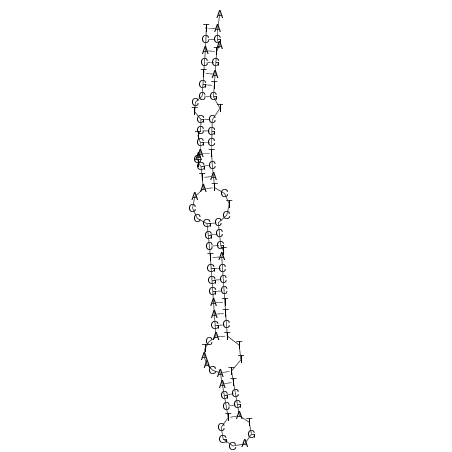

Supplement: Additional File 1 — Differential expression of known microRNAs among developmental stages. A is a pair of developmental stages in the differential expression analysis; B is the microRNA name; C and D are adult total reads; E and F are true expression levels of microRNA; G and H (∗-std) are normalized expression levels of microRNA in a developmental stage; I (fold-change (log2∗/∗)) is fold change of microRNAs in the pair of developmental stages, with negative numbers indicating downregulation and positive numbers upregulation; J is the P-value reflecting the significance of microRNA differential expression between developmental stages, whereby a smaller value indicates greater significance of the difference in microRNA expression between developmental stages and the last column (sig-label) “∗∗”: fold_change (log2) > 1 or fold_change (log2) < −1, and P < 0.01. “∗”: fold_change (log2) > 1 or fold_change (log2) < −1, and 0.01 ≤ P < 0.05. None: Others. [file Data_Sheet_1.ZIP › Addition 3-Egg/Egg-m0013.png]

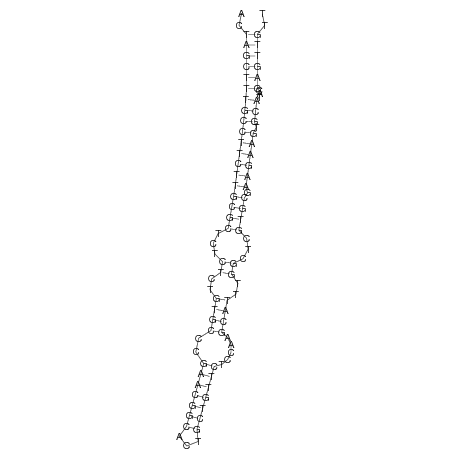

Supplement: Additional File 1 — Differential expression of known microRNAs among developmental stages. A is a pair of developmental stages in the differential expression analysis; B is the microRNA name; C and D are adult total reads; E and F are true expression levels of microRNA; G and H (∗-std) are normalized expression levels of microRNA in a developmental stage; I (fold-change (log2∗/∗)) is fold change of microRNAs in the pair of developmental stages, with negative numbers indicating downregulation and positive numbers upregulation; J is the P-value reflecting the significance of microRNA differential expression between developmental stages, whereby a smaller value indicates greater significance of the difference in microRNA expression between developmental stages and the last column (sig-label) “∗∗”: fold_change (log2) > 1 or fold_change (log2) < −1, and P < 0.01. “∗”: fold_change (log2) > 1 or fold_change (log2) < −1, and 0.01 ≤ P < 0.05. None: Others. [file Data_Sheet_1.ZIP › Addition 3-Egg/Egg-m0014.png]

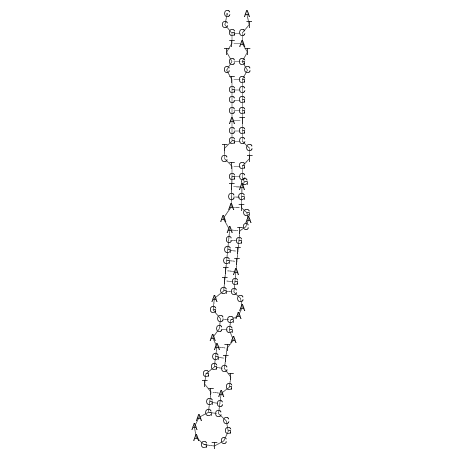

Supplement: Additional File 1 — Differential expression of known microRNAs among developmental stages. A is a pair of developmental stages in the differential expression analysis; B is the microRNA name; C and D are adult total reads; E and F are true expression levels of microRNA; G and H (∗-std) are normalized expression levels of microRNA in a developmental stage; I (fold-change (log2∗/∗)) is fold change of microRNAs in the pair of developmental stages, with negative numbers indicating downregulation and positive numbers upregulation; J is the P-value reflecting the significance of microRNA differential expression between developmental stages, whereby a smaller value indicates greater significance of the difference in microRNA expression between developmental stages and the last column (sig-label) “∗∗”: fold_change (log2) > 1 or fold_change (log2) < −1, and P < 0.01. “∗”: fold_change (log2) > 1 or fold_change (log2) < −1, and 0.01 ≤ P < 0.05. None: Others. [file Data_Sheet_1.ZIP › Addition 3-Egg/Egg-m0015.png]

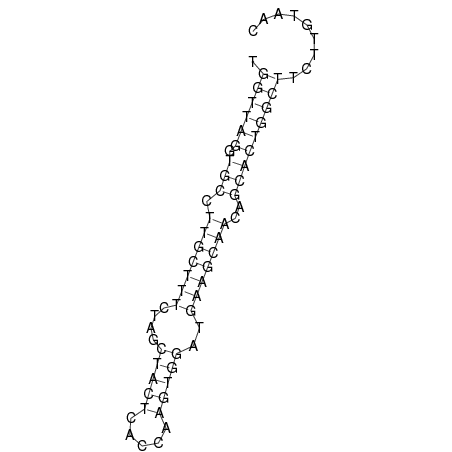

Supplement: Additional File 1 — Differential expression of known microRNAs among developmental stages. A is a pair of developmental stages in the differential expression analysis; B is the microRNA name; C and D are adult total reads; E and F are true expression levels of microRNA; G and H (∗-std) are normalized expression levels of microRNA in a developmental stage; I (fold-change (log2∗/∗)) is fold change of microRNAs in the pair of developmental stages, with negative numbers indicating downregulation and positive numbers upregulation; J is the P-value reflecting the significance of microRNA differential expression between developmental stages, whereby a smaller value indicates greater significance of the difference in microRNA expression between developmental stages and the last column (sig-label) “∗∗”: fold_change (log2) > 1 or fold_change (log2) < −1, and P < 0.01. “∗”: fold_change (log2) > 1 or fold_change (log2) < −1, and 0.01 ≤ P < 0.05. None: Others. [file Data_Sheet_1.ZIP › Addition 3-Egg/Egg-m0016.png]

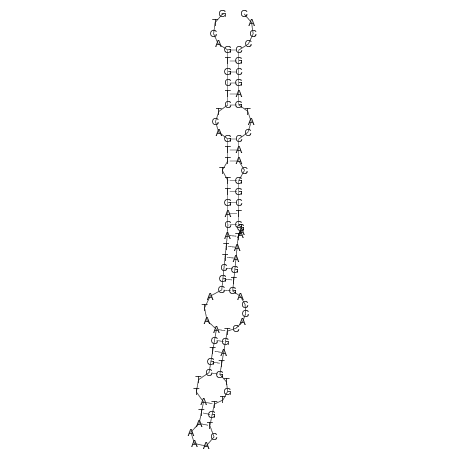

Supplement: Additional File 1 — Differential expression of known microRNAs among developmental stages. A is a pair of developmental stages in the differential expression analysis; B is the microRNA name; C and D are adult total reads; E and F are true expression levels of microRNA; G and H (∗-std) are normalized expression levels of microRNA in a developmental stage; I (fold-change (log2∗/∗)) is fold change of microRNAs in the pair of developmental stages, with negative numbers indicating downregulation and positive numbers upregulation; J is the P-value reflecting the significance of microRNA differential expression between developmental stages, whereby a smaller value indicates greater significance of the difference in microRNA expression between developmental stages and the last column (sig-label) “∗∗”: fold_change (log2) > 1 or fold_change (log2) < −1, and P < 0.01. “∗”: fold_change (log2) > 1 or fold_change (log2) < −1, and 0.01 ≤ P < 0.05. None: Others. [file Data_Sheet_1.ZIP › Addition 3-Egg/Egg-m0017.png]

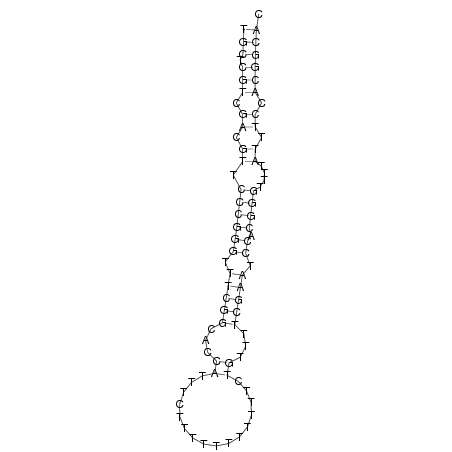

Supplement: Additional File 1 — Differential expression of known microRNAs among developmental stages. A is a pair of developmental stages in the differential expression analysis; B is the microRNA name; C and D are adult total reads; E and F are true expression levels of microRNA; G and H (∗-std) are normalized expression levels of microRNA in a developmental stage; I (fold-change (log2∗/∗)) is fold change of microRNAs in the pair of developmental stages, with negative numbers indicating downregulation and positive numbers upregulation; J is the P-value reflecting the significance of microRNA differential expression between developmental stages, whereby a smaller value indicates greater significance of the difference in microRNA expression between developmental stages and the last column (sig-label) “∗∗”: fold_change (log2) > 1 or fold_change (log2) < −1, and P < 0.01. “∗”: fold_change (log2) > 1 or fold_change (log2) < −1, and 0.01 ≤ P < 0.05. None: Others. [file Data_Sheet_1.ZIP › Addition 3-Larvae/Larvae-m0001.png]

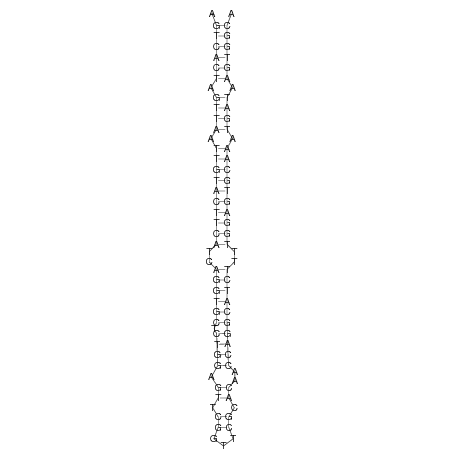

Supplement: Additional File 1 — Differential expression of known microRNAs among developmental stages. A is a pair of developmental stages in the differential expression analysis; B is the microRNA name; C and D are adult total reads; E and F are true expression levels of microRNA; G and H (∗-std) are normalized expression levels of microRNA in a developmental stage; I (fold-change (log2∗/∗)) is fold change of microRNAs in the pair of developmental stages, with negative numbers indicating downregulation and positive numbers upregulation; J is the P-value reflecting the significance of microRNA differential expression between developmental stages, whereby a smaller value indicates greater significance of the difference in microRNA expression between developmental stages and the last column (sig-label) “∗∗”: fold_change (log2) > 1 or fold_change (log2) < −1, and P < 0.01. “∗”: fold_change (log2) > 1 or fold_change (log2) < −1, and 0.01 ≤ P < 0.05. None: Others. [file Data_Sheet_1.ZIP › Addition 3-Larvae/Larvae-m0003.png]

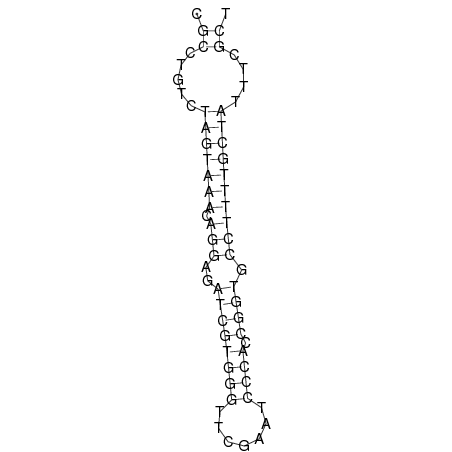

Supplement: Additional File 1 — Differential expression of known microRNAs among developmental stages. A is a pair of developmental stages in the differential expression analysis; B is the microRNA name; C and D are adult total reads; E and F are true expression levels of microRNA; G and H (∗-std) are normalized expression levels of microRNA in a developmental stage; I (fold-change (log2∗/∗)) is fold change of microRNAs in the pair of developmental stages, with negative numbers indicating downregulation and positive numbers upregulation; J is the P-value reflecting the significance of microRNA differential expression between developmental stages, whereby a smaller value indicates greater significance of the difference in microRNA expression between developmental stages and the last column (sig-label) “∗∗”: fold_change (log2) > 1 or fold_change (log2) < −1, and P < 0.01. “∗”: fold_change (log2) > 1 or fold_change (log2) < −1, and 0.01 ≤ P < 0.05. None: Others. [file Data_Sheet_1.ZIP › Addition 3-Larvae/Larvae-m0004.png]

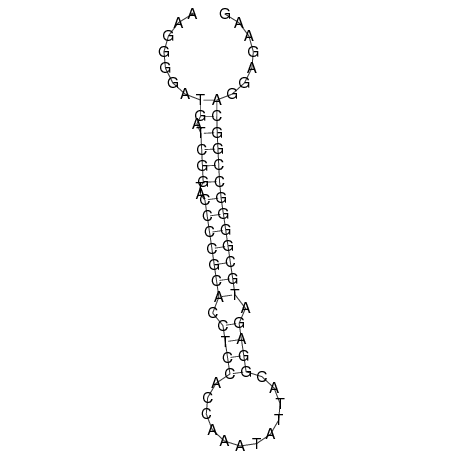

Supplement: Additional File 1 — Differential expression of known microRNAs among developmental stages. A is a pair of developmental stages in the differential expression analysis; B is the microRNA name; C and D are adult total reads; E and F are true expression levels of microRNA; G and H (∗-std) are normalized expression levels of microRNA in a developmental stage; I (fold-change (log2∗/∗)) is fold change of microRNAs in the pair of developmental stages, with negative numbers indicating downregulation and positive numbers upregulation; J is the P-value reflecting the significance of microRNA differential expression between developmental stages, whereby a smaller value indicates greater significance of the difference in microRNA expression between developmental stages and the last column (sig-label) “∗∗”: fold_change (log2) > 1 or fold_change (log2) < −1, and P < 0.01. “∗”: fold_change (log2) > 1 or fold_change (log2) < −1, and 0.01 ≤ P < 0.05. None: Others. [file Data_Sheet_1.ZIP › Addition 3-Larvae/Larvae-m0007.png]

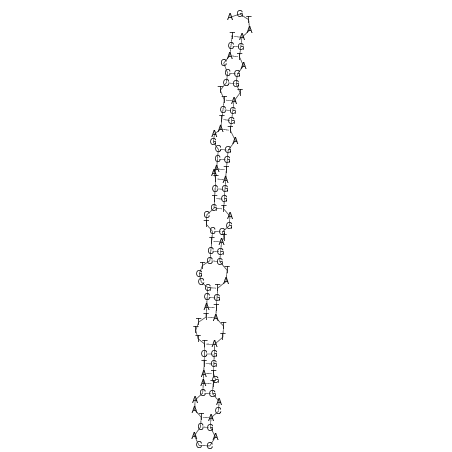

Supplement: Additional File 1 — Differential expression of known microRNAs among developmental stages. A is a pair of developmental stages in the differential expression analysis; B is the microRNA name; C and D are adult total reads; E and F are true expression levels of microRNA; G and H (∗-std) are normalized expression levels of microRNA in a developmental stage; I (fold-change (log2∗/∗)) is fold change of microRNAs in the pair of developmental stages, with negative numbers indicating downregulation and positive numbers upregulation; J is the P-value reflecting the significance of microRNA differential expression between developmental stages, whereby a smaller value indicates greater significance of the difference in microRNA expression between developmental stages and the last column (sig-label) “∗∗”: fold_change (log2) > 1 or fold_change (log2) < −1, and P < 0.01. “∗”: fold_change (log2) > 1 or fold_change (log2) < −1, and 0.01 ≤ P < 0.05. None: Others. [file Data_Sheet_1.ZIP › Addition 3-Larvae/Larvae-m0008.png]

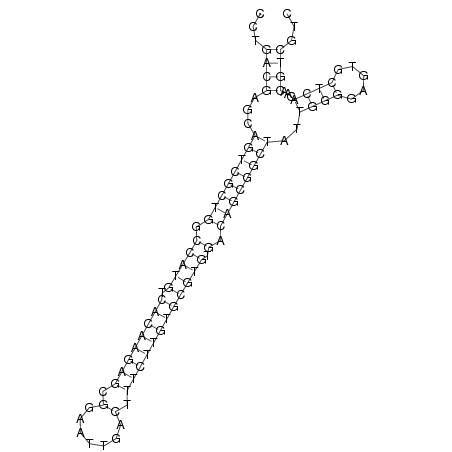

Supplement: Additional File 1 — Differential expression of known microRNAs among developmental stages. A is a pair of developmental stages in the differential expression analysis; B is the microRNA name; C and D are adult total reads; E and F are true expression levels of microRNA; G and H (∗-std) are normalized expression levels of microRNA in a developmental stage; I (fold-change (log2∗/∗)) is fold change of microRNAs in the pair of developmental stages, with negative numbers indicating downregulation and positive numbers upregulation; J is the P-value reflecting the significance of microRNA differential expression between developmental stages, whereby a smaller value indicates greater significance of the difference in microRNA expression between developmental stages and the last column (sig-label) “∗∗”: fold_change (log2) > 1 or fold_change (log2) < −1, and P < 0.01. “∗”: fold_change (log2) > 1 or fold_change (log2) < −1, and 0.01 ≤ P < 0.05. None: Others. [file Data_Sheet_1.ZIP › Addition 3-Larvae/Larvae-m0009.png]

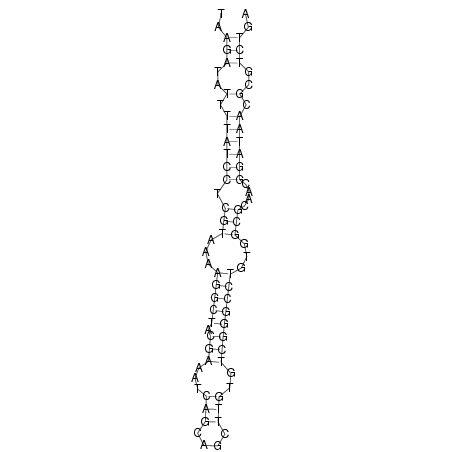

Supplement: Additional File 1 — Differential expression of known microRNAs among developmental stages. A is a pair of developmental stages in the differential expression analysis; B is the microRNA name; C and D are adult total reads; E and F are true expression levels of microRNA; G and H (∗-std) are normalized expression levels of microRNA in a developmental stage; I (fold-change (log2∗/∗)) is fold change of microRNAs in the pair of developmental stages, with negative numbers indicating downregulation and positive numbers upregulation; J is the P-value reflecting the significance of microRNA differential expression between developmental stages, whereby a smaller value indicates greater significance of the difference in microRNA expression between developmental stages and the last column (sig-label) “∗∗”: fold_change (log2) > 1 or fold_change (log2) < −1, and P < 0.01. “∗”: fold_change (log2) > 1 or fold_change (log2) < −1, and 0.01 ≤ P < 0.05. None: Others. [file Data_Sheet_1.ZIP › Addition 3-Larvae/Larvae-m0010.png]

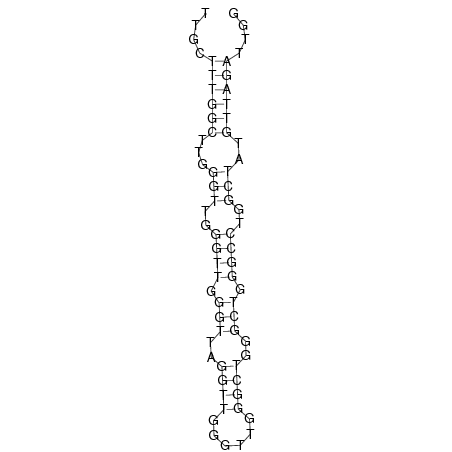

Supplement: Additional File 1 — Differential expression of known microRNAs among developmental stages. A is a pair of developmental stages in the differential expression analysis; B is the microRNA name; C and D are adult total reads; E and F are true expression levels of microRNA; G and H (∗-std) are normalized expression levels of microRNA in a developmental stage; I (fold-change (log2∗/∗)) is fold change of microRNAs in the pair of developmental stages, with negative numbers indicating downregulation and positive numbers upregulation; J is the P-value reflecting the significance of microRNA differential expression between developmental stages, whereby a smaller value indicates greater significance of the difference in microRNA expression between developmental stages and the last column (sig-label) “∗∗”: fold_change (log2) > 1 or fold_change (log2) < −1, and P < 0.01. “∗”: fold_change (log2) > 1 or fold_change (log2) < −1, and 0.01 ≤ P < 0.05. None: Others. [file Data_Sheet_1.ZIP › Addition 3-Larvae/Larvae-m0011.png]

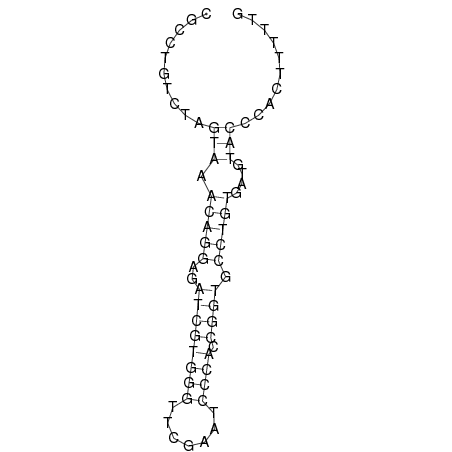

Supplement: Additional File 1 — Differential expression of known microRNAs among developmental stages. A is a pair of developmental stages in the differential expression analysis; B is the microRNA name; C and D are adult total reads; E and F are true expression levels of microRNA; G and H (∗-std) are normalized expression levels of microRNA in a developmental stage; I (fold-change (log2∗/∗)) is fold change of microRNAs in the pair of developmental stages, with negative numbers indicating downregulation and positive numbers upregulation; J is the P-value reflecting the significance of microRNA differential expression between developmental stages, whereby a smaller value indicates greater significance of the difference in microRNA expression between developmental stages and the last column (sig-label) “∗∗”: fold_change (log2) > 1 or fold_change (log2) < −1, and P < 0.01. “∗”: fold_change (log2) > 1 or fold_change (log2) < −1, and 0.01 ≤ P < 0.05. None: Others. [file Data_Sheet_1.ZIP › Addition 3-Larvae/Larvae-m0013.png]

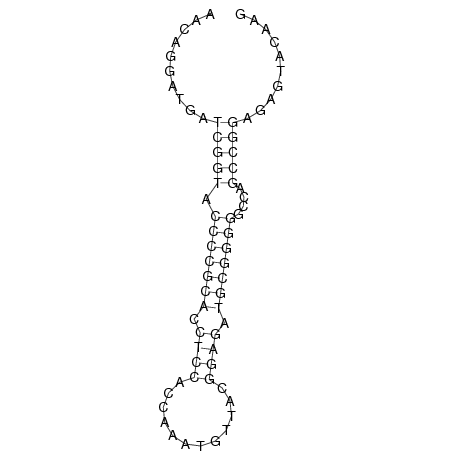

Supplement: Additional File 1 — Differential expression of known microRNAs among developmental stages. A is a pair of developmental stages in the differential expression analysis; B is the microRNA name; C and D are adult total reads; E and F are true expression levels of microRNA; G and H (∗-std) are normalized expression levels of microRNA in a developmental stage; I (fold-change (log2∗/∗)) is fold change of microRNAs in the pair of developmental stages, with negative numbers indicating downregulation and positive numbers upregulation; J is the P-value reflecting the significance of microRNA differential expression between developmental stages, whereby a smaller value indicates greater significance of the difference in microRNA expression between developmental stages and the last column (sig-label) “∗∗”: fold_change (log2) > 1 or fold_change (log2) < −1, and P < 0.01. “∗”: fold_change (log2) > 1 or fold_change (log2) < −1, and 0.01 ≤ P < 0.05. None: Others. [file Data_Sheet_1.ZIP › Addition 3-Larvae/Larvae-m0014.png]

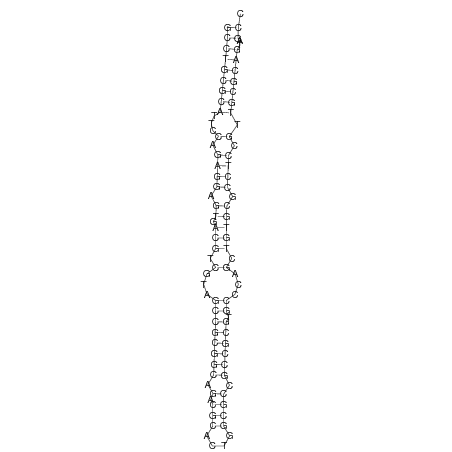

Supplement: Additional File 1 — Differential expression of known microRNAs among developmental stages. A is a pair of developmental stages in the differential expression analysis; B is the microRNA name; C and D are adult total reads; E and F are true expression levels of microRNA; G and H (∗-std) are normalized expression levels of microRNA in a developmental stage; I (fold-change (log2∗/∗)) is fold change of microRNAs in the pair of developmental stages, with negative numbers indicating downregulation and positive numbers upregulation; J is the P-value reflecting the significance of microRNA differential expression between developmental stages, whereby a smaller value indicates greater significance of the difference in microRNA expression between developmental stages and the last column (sig-label) “∗∗”: fold_change (log2) > 1 or fold_change (log2) < −1, and P < 0.01. “∗”: fold_change (log2) > 1 or fold_change (log2) < −1, and 0.01 ≤ P < 0.05. None: Others. [file Data_Sheet_1.ZIP › Addition 3-Larvae/Larvae-m0015.png]

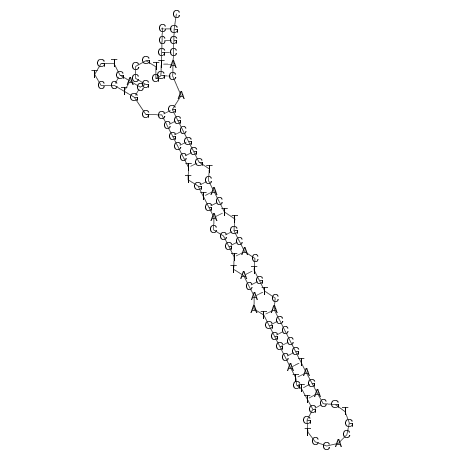

Supplement: Additional File 1 — Differential expression of known microRNAs among developmental stages. A is a pair of developmental stages in the differential expression analysis; B is the microRNA name; C and D are adult total reads; E and F are true expression levels of microRNA; G and H (∗-std) are normalized expression levels of microRNA in a developmental stage; I (fold-change (log2∗/∗)) is fold change of microRNAs in the pair of developmental stages, with negative numbers indicating downregulation and positive numbers upregulation; J is the P-value reflecting the significance of microRNA differential expression between developmental stages, whereby a smaller value indicates greater significance of the difference in microRNA expression between developmental stages and the last column (sig-label) “∗∗”: fold_change (log2) > 1 or fold_change (log2) < −1, and P < 0.01. “∗”: fold_change (log2) > 1 or fold_change (log2) < −1, and 0.01 ≤ P < 0.05. None: Others. [file Data_Sheet_1.ZIP › Addition 3-Larvae/Larvae-m0016.png]

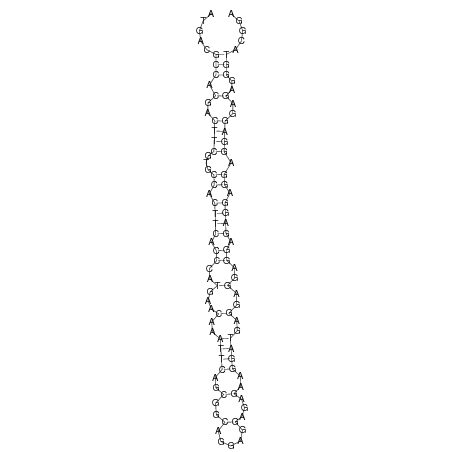

Supplement: Additional File 1 — Differential expression of known microRNAs among developmental stages. A is a pair of developmental stages in the differential expression analysis; B is the microRNA name; C and D are adult total reads; E and F are true expression levels of microRNA; G and H (∗-std) are normalized expression levels of microRNA in a developmental stage; I (fold-change (log2∗/∗)) is fold change of microRNAs in the pair of developmental stages, with negative numbers indicating downregulation and positive numbers upregulation; J is the P-value reflecting the significance of microRNA differential expression between developmental stages, whereby a smaller value indicates greater significance of the difference in microRNA expression between developmental stages and the last column (sig-label) “∗∗”: fold_change (log2) > 1 or fold_change (log2) < −1, and P < 0.01. “∗”: fold_change (log2) > 1 or fold_change (log2) < −1, and 0.01 ≤ P < 0.05. None: Others. [file Data_Sheet_1.ZIP › Addition 3-Larvae/Larvae-m0017.png]

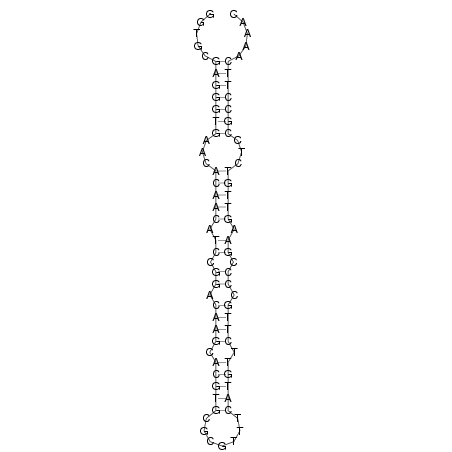

Supplement: Additional File 1 — Differential expression of known microRNAs among developmental stages. A is a pair of developmental stages in the differential expression analysis; B is the microRNA name; C and D are adult total reads; E and F are true expression levels of microRNA; G and H (∗-std) are normalized expression levels of microRNA in a developmental stage; I (fold-change (log2∗/∗)) is fold change of microRNAs in the pair of developmental stages, with negative numbers indicating downregulation and positive numbers upregulation; J is the P-value reflecting the significance of microRNA differential expression between developmental stages, whereby a smaller value indicates greater significance of the difference in microRNA expression between developmental stages and the last column (sig-label) “∗∗”: fold_change (log2) > 1 or fold_change (log2) < −1, and P < 0.01. “∗”: fold_change (log2) > 1 or fold_change (log2) < −1, and 0.01 ≤ P < 0.05. None: Others. [file Data_Sheet_1.ZIP › Addition 3-Larvae/Larvae-m0018.png]

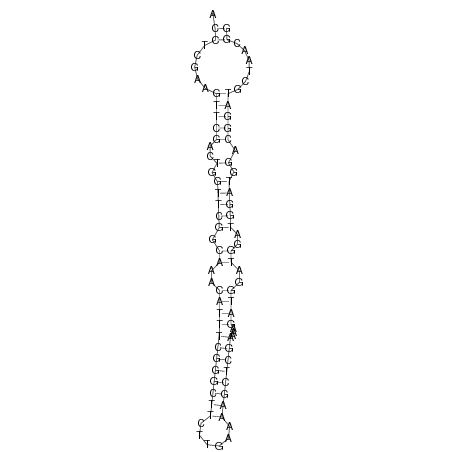

Supplement: Additional File 1 — Differential expression of known microRNAs among developmental stages. A is a pair of developmental stages in the differential expression analysis; B is the microRNA name; C and D are adult total reads; E and F are true expression levels of microRNA; G and H (∗-std) are normalized expression levels of microRNA in a developmental stage; I (fold-change (log2∗/∗)) is fold change of microRNAs in the pair of developmental stages, with negative numbers indicating downregulation and positive numbers upregulation; J is the P-value reflecting the significance of microRNA differential expression between developmental stages, whereby a smaller value indicates greater significance of the difference in microRNA expression between developmental stages and the last column (sig-label) “∗∗”: fold_change (log2) > 1 or fold_change (log2) < −1, and P < 0.01. “∗”: fold_change (log2) > 1 or fold_change (log2) < −1, and 0.01 ≤ P < 0.05. None: Others. [file Data_Sheet_1.ZIP › Addition 3-Larvae/Larvae-m0019.png]

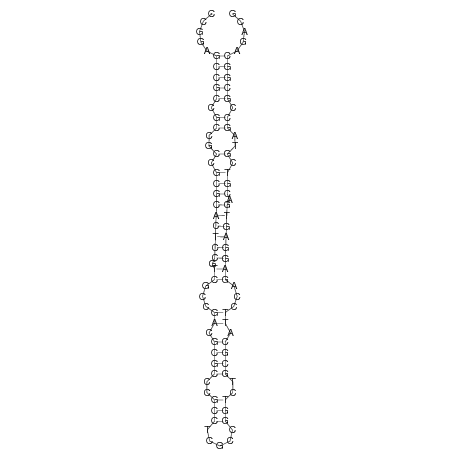

Supplement: Additional File 1 — Differential expression of known microRNAs among developmental stages. A is a pair of developmental stages in the differential expression analysis; B is the microRNA name; C and D are adult total reads; E and F are true expression levels of microRNA; G and H (∗-std) are normalized expression levels of microRNA in a developmental stage; I (fold-change (log2∗/∗)) is fold change of microRNAs in the pair of developmental stages, with negative numbers indicating downregulation and positive numbers upregulation; J is the P-value reflecting the significance of microRNA differential expression between developmental stages, whereby a smaller value indicates greater significance of the difference in microRNA expression between developmental stages and the last column (sig-label) “∗∗”: fold_change (log2) > 1 or fold_change (log2) < −1, and P < 0.01. “∗”: fold_change (log2) > 1 or fold_change (log2) < −1, and 0.01 ≤ P < 0.05. None: Others. [file Data_Sheet_1.ZIP › Addition 3-Larvae/Larvae-m0020.png]

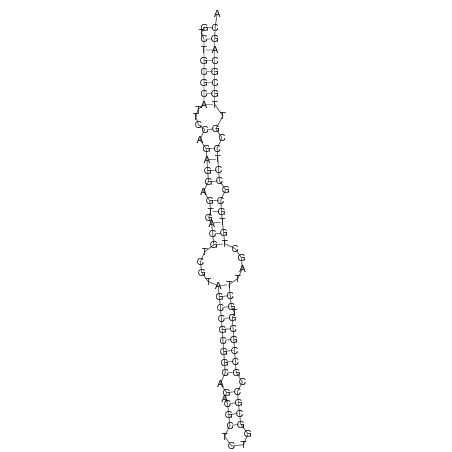

Supplement: Additional File 1 — Differential expression of known microRNAs among developmental stages. A is a pair of developmental stages in the differential expression analysis; B is the microRNA name; C and D are adult total reads; E and F are true expression levels of microRNA; G and H (∗-std) are normalized expression levels of microRNA in a developmental stage; I (fold-change (log2∗/∗)) is fold change of microRNAs in the pair of developmental stages, with negative numbers indicating downregulation and positive numbers upregulation; J is the P-value reflecting the significance of microRNA differential expression between developmental stages, whereby a smaller value indicates greater significance of the difference in microRNA expression between developmental stages and the last column (sig-label) “∗∗”: fold_change (log2) > 1 or fold_change (log2) < −1, and P < 0.01. “∗”: fold_change (log2) > 1 or fold_change (log2) < −1, and 0.01 ≤ P < 0.05. None: Others. [file Data_Sheet_1.ZIP › Addition 3-Larvae/Larvae-m0021.png]

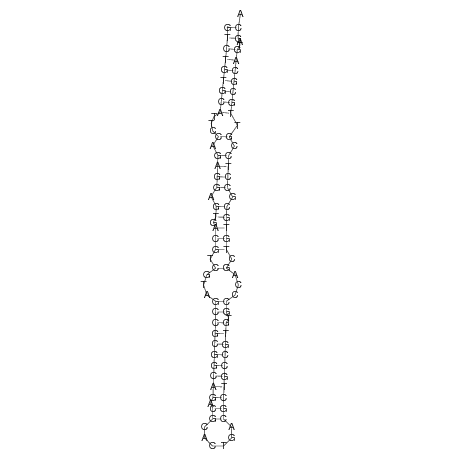

Supplement: Additional File 1 — Differential expression of known microRNAs among developmental stages. A is a pair of developmental stages in the differential expression analysis; B is the microRNA name; C and D are adult total reads; E and F are true expression levels of microRNA; G and H (∗-std) are normalized expression levels of microRNA in a developmental stage; I (fold-change (log2∗/∗)) is fold change of microRNAs in the pair of developmental stages, with negative numbers indicating downregulation and positive numbers upregulation; J is the P-value reflecting the significance of microRNA differential expression between developmental stages, whereby a smaller value indicates greater significance of the difference in microRNA expression between developmental stages and the last column (sig-label) “∗∗”: fold_change (log2) > 1 or fold_change (log2) < −1, and P < 0.01. “∗”: fold_change (log2) > 1 or fold_change (log2) < −1, and 0.01 ≤ P < 0.05. None: Others. [file Data_Sheet_1.ZIP › Addition 3-Larvae/Larvae-m0022.png]

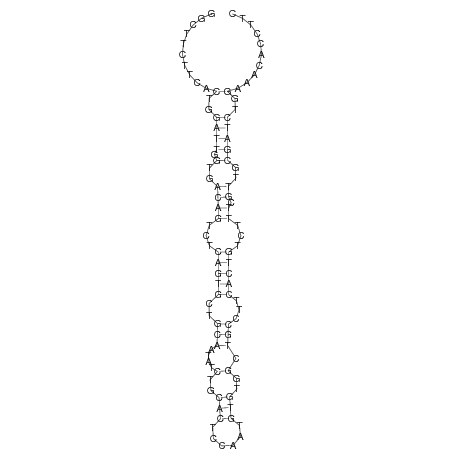

Supplement: Additional File 1 — Differential expression of known microRNAs among developmental stages. A is a pair of developmental stages in the differential expression analysis; B is the microRNA name; C and D are adult total reads; E and F are true expression levels of microRNA; G and H (∗-std) are normalized expression levels of microRNA in a developmental stage; I (fold-change (log2∗/∗)) is fold change of microRNAs in the pair of developmental stages, with negative numbers indicating downregulation and positive numbers upregulation; J is the P-value reflecting the significance of microRNA differential expression between developmental stages, whereby a smaller value indicates greater significance of the difference in microRNA expression between developmental stages and the last column (sig-label) “∗∗”: fold_change (log2) > 1 or fold_change (log2) < −1, and P < 0.01. “∗”: fold_change (log2) > 1 or fold_change (log2) < −1, and 0.01 ≤ P < 0.05. None: Others. [file Data_Sheet_1.ZIP › Addition 3-Larvae/Larvae-m0023.png]

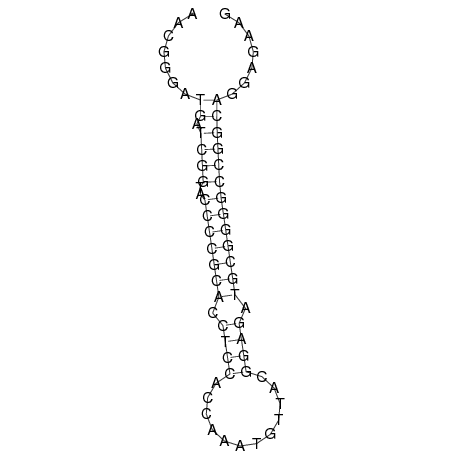

Supplement: Additional File 1 — Differential expression of known microRNAs among developmental stages. A is a pair of developmental stages in the differential expression analysis; B is the microRNA name; C and D are adult total reads; E and F are true expression levels of microRNA; G and H (∗-std) are normalized expression levels of microRNA in a developmental stage; I (fold-change (log2∗/∗)) is fold change of microRNAs in the pair of developmental stages, with negative numbers indicating downregulation and positive numbers upregulation; J is the P-value reflecting the significance of microRNA differential expression between developmental stages, whereby a smaller value indicates greater significance of the difference in microRNA expression between developmental stages and the last column (sig-label) “∗∗”: fold_change (log2) > 1 or fold_change (log2) < −1, and P < 0.01. “∗”: fold_change (log2) > 1 or fold_change (log2) < −1, and 0.01 ≤ P < 0.05. None: Others. [file Data_Sheet_1.ZIP › Addition 3-Larvae/Larvae-m0024.png]

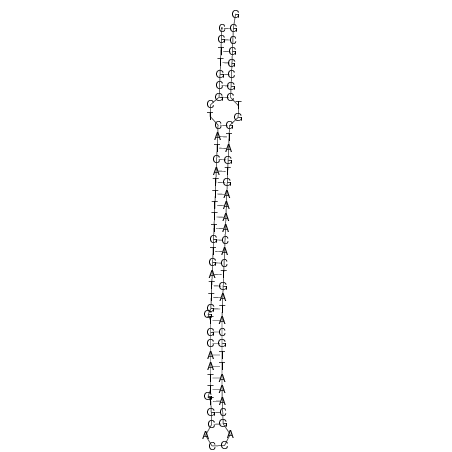

Supplement: Additional File 1 — Differential expression of known microRNAs among developmental stages. A is a pair of developmental stages in the differential expression analysis; B is the microRNA name; C and D are adult total reads; E and F are true expression levels of microRNA; G and H (∗-std) are normalized expression levels of microRNA in a developmental stage; I (fold-change (log2∗/∗)) is fold change of microRNAs in the pair of developmental stages, with negative numbers indicating downregulation and positive numbers upregulation; J is the P-value reflecting the significance of microRNA differential expression between developmental stages, whereby a smaller value indicates greater significance of the difference in microRNA expression between developmental stages and the last column (sig-label) “∗∗”: fold_change (log2) > 1 or fold_change (log2) < −1, and P < 0.01. “∗”: fold_change (log2) > 1 or fold_change (log2) < −1, and 0.01 ≤ P < 0.05. None: Others. [file Data_Sheet_1.ZIP › Addition 3-Larvae/Larvae-m0025.png]

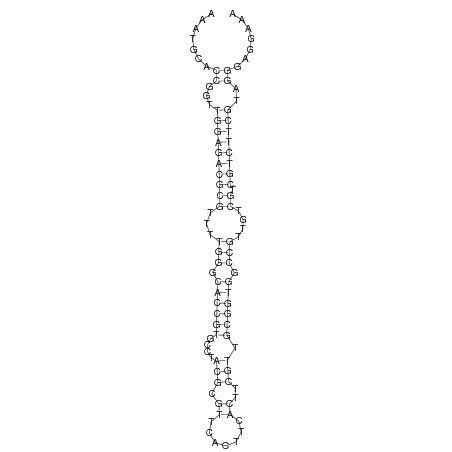

Supplement: Additional File 1 — Differential expression of known microRNAs among developmental stages. A is a pair of developmental stages in the differential expression analysis; B is the microRNA name; C and D are adult total reads; E and F are true expression levels of microRNA; G and H (∗-std) are normalized expression levels of microRNA in a developmental stage; I (fold-change (log2∗/∗)) is fold change of microRNAs in the pair of developmental stages, with negative numbers indicating downregulation and positive numbers upregulation; J is the P-value reflecting the significance of microRNA differential expression between developmental stages, whereby a smaller value indicates greater significance of the difference in microRNA expression between developmental stages and the last column (sig-label) “∗∗”: fold_change (log2) > 1 or fold_change (log2) < −1, and P < 0.01. “∗”: fold_change (log2) > 1 or fold_change (log2) < −1, and 0.01 ≤ P < 0.05. None: Others. [file Data_Sheet_1.ZIP › Addition 3-Nymph/Nymph-m0007.png]

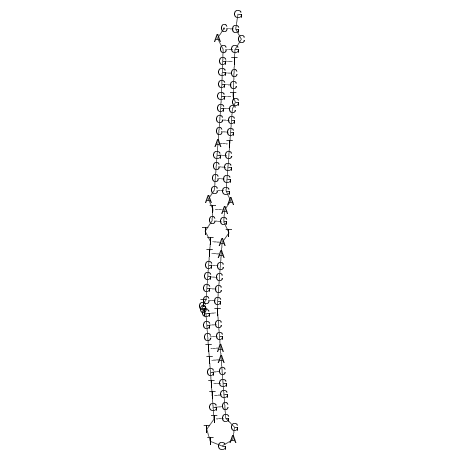

Supplement: Additional File 1 — Differential expression of known microRNAs among developmental stages. A is a pair of developmental stages in the differential expression analysis; B is the microRNA name; C and D are adult total reads; E and F are true expression levels of microRNA; G and H (∗-std) are normalized expression levels of microRNA in a developmental stage; I (fold-change (log2∗/∗)) is fold change of microRNAs in the pair of developmental stages, with negative numbers indicating downregulation and positive numbers upregulation; J is the P-value reflecting the significance of microRNA differential expression between developmental stages, whereby a smaller value indicates greater significance of the difference in microRNA expression between developmental stages and the last column (sig-label) “∗∗”: fold_change (log2) > 1 or fold_change (log2) < −1, and P < 0.01. “∗”: fold_change (log2) > 1 or fold_change (log2) < −1, and 0.01 ≤ P < 0.05. None: Others. [file Data_Sheet_1.ZIP › Addition 3-Nymph/Nymph-m0009.png]

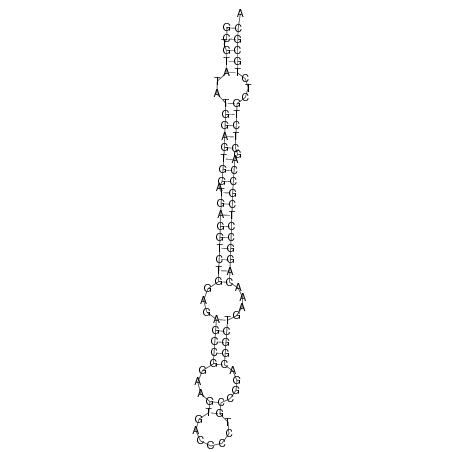

Supplement: Additional File 1 — Differential expression of known microRNAs among developmental stages. A is a pair of developmental stages in the differential expression analysis; B is the microRNA name; C and D are adult total reads; E and F are true expression levels of microRNA; G and H (∗-std) are normalized expression levels of microRNA in a developmental stage; I (fold-change (log2∗/∗)) is fold change of microRNAs in the pair of developmental stages, with negative numbers indicating downregulation and positive numbers upregulation; J is the P-value reflecting the significance of microRNA differential expression between developmental stages, whereby a smaller value indicates greater significance of the difference in microRNA expression between developmental stages and the last column (sig-label) “∗∗”: fold_change (log2) > 1 or fold_change (log2) < −1, and P < 0.01. “∗”: fold_change (log2) > 1 or fold_change (log2) < −1, and 0.01 ≤ P < 0.05. None: Others. [file Data_Sheet_1.ZIP › Addition 3-Nymph/Nymph-m0010.png]

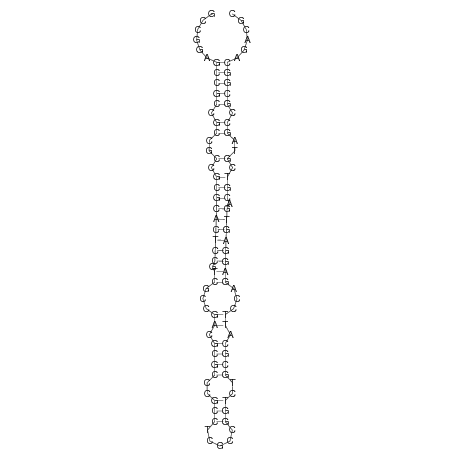

Supplement: Additional File 1 — Differential expression of known microRNAs among developmental stages. A is a pair of developmental stages in the differential expression analysis; B is the microRNA name; C and D are adult total reads; E and F are true expression levels of microRNA; G and H (∗-std) are normalized expression levels of microRNA in a developmental stage; I (fold-change (log2∗/∗)) is fold change of microRNAs in the pair of developmental stages, with negative numbers indicating downregulation and positive numbers upregulation; J is the P-value reflecting the significance of microRNA differential expression between developmental stages, whereby a smaller value indicates greater significance of the difference in microRNA expression between developmental stages and the last column (sig-label) “∗∗”: fold_change (log2) > 1 or fold_change (log2) < −1, and P < 0.01. “∗”: fold_change (log2) > 1 or fold_change (log2) < −1, and 0.01 ≤ P < 0.05. None: Others. [file Data_Sheet_1.ZIP › Addition 3-Nymph/Nymph-m0011.png]
